# Supplementary material for: Intervention development and treatment success in UK health technology assessment funded trials of physical rehabilitation: a mixed methods analysis
Source: BMJ Open. 2019 Aug 28;9(8):e026289. doi: 10.1136/bmjopen-2018-026289 (PMC6720467; doi:10.1136/bmjopen-2018-026289)
Supplement: Author's manuscript [file bmjopen-2018-026289.draft_revisions.pdf]

BMJ Open is committed to open peer review. As part of this commitment we make the peer review history of every article we publish publicly available.

When an article is published we post the peer reviewers' comments and the authors' responses online. We also post the versions of the paper that were used during peer review. These are the versions that the peer review comments apply to.

The versions of the paper that follow are the versions that were submitted during the peer review process. They are not the versions of record or the final published versions. They should not be cited or distributed as the published version of this manuscript.

BMJ Open is an open access journal and the full, final, typeset and author-corrected version of record of the manuscript is available on our site with no access controls, subscription charges or pay-per-view fees (http://bmjopen.bmj.com).

If you have any questions on BMJ Open's open peer review process please email info.bmjopen@bmj.com

BMJ Open

Intervention development and treatment success in UK Health Technology Assessment funded trials of rehabilitation: a mixed methods analysis

Journal:	*BMJ Open*
Manuscript ID	bmjopen-2018-026289
Article Type:	Research
Date Submitted by the Author:	24-Aug-2018

Complete List of Authors:	Goodwin, Victoria; University of Exeter Medical School<br>Hill, Jacqueline; University of Exeter Medical School<br>Fullam, James; University of Exeter Medical School<br>Finning, Katie; University of Exeter Medical School<br>Pentecost, C; University of Exeter Medical School<br>Richards, David; University of Exeter, Medical School

Keywords:	rehabilitation, intervention development, mixed methods, randomised controlled trials

SCHOLARONE™
Manuscripts

For peer review only

Intervention development and treatment success in UK Health Technology Assessment funded trials of rehabilitation: a mixed methods analysis

*Victoria A Goodwin, University of Exeter Medical School, Exeter UK

Jacqueline J Hill, University of Exeter Medical School, Exeter UK

James Fullam, University of Exeter Medical School, Exeter UK

Katie Finning, University of Exeter Medical School, Exeter UK

Claire Pentecost, University of Exeter Medical School, Exeter UK

David A Richards, University of Exeter Medical School, Exeter UK

* Corresponding author: 2.26 South Cloisters, University of Exeter Medical School, St Luke's Campus, Magdalen Road, Exeter EX1 2LU. Telephone 01392 722745 v.goodwin@exeter.ac.uk

Word count: 2607

Keywords

Rehabilitation, randomised controlled trials, quality, intervention development, mixed methods

For peer review only

Abstract

Objectives: Rehabilitation is a complex process and trials of rehabilitation interventions are increasing in number. This study aimed to establish treatment success rates in rehabilitation trials funded by the NIHR Health Technology Assessment (HTA) programme and examine any relationship between treatment success and the quality of intervention development work undertaken.

Design: Mixed methods study

Setting: UK

Methods:

The NIHR HTA portfolio was searched for all completed definitive randomised controlled trials of physiotherapy, occupational therapy or speech and language therapy from inception to July 2016. Treatment success was categorised according to criteria developed by Djulbegovic and colleagues. Detailed textual data regarding any intervention development work were extracted from the trial reports and supporting publications and informed the development of a quality rating. Mixed methods integrative analysis was undertaken to explore the relationship between the quantitative and qualitative data using joint displays.

Results: Fifteen trials were included in the review. Of these, five reported a definitive finding, four of which were in favour of the 'new' intervention. Eight trials reported a true negative (no difference) outcome. Integrative analysis indicated those with lower quality intervention development work were less likely to report treatment success, although some older and possibly less well reported trials reported effective interventions.

Conclusions: Despite much effort and funding, most rehabilitation trials report equivocal findings. Greater focus on high quality intervention development may reduce the likelihood of a null result in the definitive trial.

For peer review only

Strengths and limitations of this study:

- The use of mixed methods integrative analyses to explore the relationship between quality of intervention development work and treatment success.
- The study comprised randomised controlled trials of rehabilitation from a single UK funder.
- Factors other than intervention development can influence treatment success

For peer review only - http://bmjopen.bmj.com/site/about/guidelines.xhtml

BACKGROUND

Rehabilitation is *"a set of interventions designed to optimise function and reduce disability in individuals with health conditions in interaction with their environment".*[1] and is an essential aspect of healthcare provision. By its very nature rehabilitation in clinical practice is an individually focused, complex activity, involving interventions that are multi-faceted and often implicit in nature[2] and as such, historically, this has been viewed as a barrier to undertaking research.[3] This said, there is a growing body of evidence from randomised controlled trials of rehabilitation, suggesting that these challenges can be overcome.[4] This may, in part, be supported by the publication of the MRC Framework for developing and evaluating complex interventions.[5 6]

The framework was developed to optimise the likelihood that new interventions are not rejected as being ineffective when inadequate effort has been made in the development of the intervention.[7] Likewise, Chalmers and Glasziou[8] highlighted the importance of avoiding research waste and recommended that sufficient effort is made to ensure the relevant research questions are identified and addressed using high quality research methods. However, there appears to have been no formal evaluation of the impact of using the development component of the framework on trial outcomes and whether we are observing evidence of effective interventions being developed.

Previous UK[9] and USA[10] reviews synthesised successful and non-successful treatment outcomes from trials of new interventions in order to assess the equipoise principle and to understand what return has been achieved on the investment made by those taking part in the trials, researchers and funders. Dent and Raftery[9] reported 24% (20/85) primary outcome comparisons as having a positive result, of which 16/85 (19%) were in favour of the new intervention, with 19/85 (22%) comparisons reporting a true negative outcome. However, these authors did not focus on rehabilitation interventions, nor did they seek to understand factors that may impact on treatment success, such as the quality or intensity of intervention development pre-trial procedures. In order to build on the work of Dent and Raftery,[9] who evaluated the outcomes of UK National Institute of Health Research (NIHR) Health Technology Assessment (HTA) funded research, we aimed to use data,[11] from the same funding stream to: (1) establish the treatment outcomes of NIHR HTA funded randomised controlled trials of physiotherapy, occupational therapy and speech and language therapy using Djulbegovic's classification[10]; (2) establish how many new interventions were found to be effective; (3) examine what work had been done in terms of developing the new intervention; and (4) examine the relationship between (1) and (3). We adopted a mixed methods approach to address the study aims. Although evidence of using integrative mixed methods approaches in synthesising evidence on complex interventions is limited, mixing together qualitative and quantitative data can generate understanding that has the potential to be greater than the sum of the individual parts.[12]

METHODS

Design

We undertook a review of NIHR HTA funded randomised controlled trials of rehabilitation interventions using narrative synthesis of outcomes and mixed methods analysis of the relationship between intervention development and categorical treatment outcomes using joint displays.

Data sources and inclusion criteria

For peer review only - http://bmjopen.bmj.com/site/about/guidelines.xhtml

We included superiority randomised controlled trials of physiotherapy, occupational therapy, or speech and language therapy funded by the NIHR HTA programme. The NIHR HTA programme is the leading public funding source for randomised controlled trials (RCTs) in the UK and trials of rehabilitation are increasingly part of the portfolio. We only included completed RCTs whose main trial findings were reported in an HTA monograph or peer-reviewed publication. We excluded pilot or feasibility RCTs and systematic reviews, along with studies where the interventions were primarily psychological or cognitive, those where it was unclear which study arm was the control, where there was a lack of a clear primary outcome (including primary time point) or where the primary outcome findings were not reported with a 95% confidence interval.

Search and screening

We searched the HTA Project Portfolio (since superseded by the NIHR Journals Library) from inception to July 2016 using the following keywords: physiotherap*, occupational therap*, speech and language therap* and rehabilitation. We removed duplicates and then titles and scientific abstracts were reviewed for potential inclusion by one person and checked by a second. Subsequently full text reports were screened for inclusion by one person and checked by a second. Any disagreements were discussed and agreed with a third person.

Data extraction

All data were extracted by one person and checked by a second. Discrepancies were discussed and resolved with a third person.

Trial data: Data extracted from each trial publication included trial design, target population, primary outcome(s) and time point, minimal important clinical difference (MCID) that the trial aimed to detect, planned and achieved sample size, and primary outcome results with 95% CI. We also recorded the professional background of the Chief Investigator and amount of funding awarded.

Intervention development data: Using the revised version of Criteria for Reporting the Development and Evaluation of Complex Interventions (CReDECI 2)[13] and the Template for Intervention Description and Replication checklist (TIDieR)[14] as frameworks we extracted all available documentary (qualitative) data from the body of the text regarding intervention development, including descriptions of underlying theory, intervention components and reasons for selection, intended interactions between components, contextual considerations, piloting of intervention and impact of definitive intervention to be evaluated, control components, planned intervention delivery and materials. Where additional supporting publications were cited, such as a protocol or intervention development studies, we used these as additional sources of documentary data.

Data analysis

We used summary statistics to describe the characteristics of the included studies. We categorised primary outcome findings into one of six treatment outcome categories as described by Djulbegovic and colleagues,[10] these being: 1) statistically significant in favour of the new treatment, 2) statistically significant in favour of the control treatment 3) true negative, 4) truly inconclusive, 5) inconclusive in favour of new treatment or 6) inconclusive in favour of the control treatment. This was achieved by comparing the 95% confidence interval for the difference in primary outcome to the difference specified in the sample size calculation.[9] If the 95% confidence interval excluded a

For peer review only - http://bmjopen.bmj.com/site/about/guidelines.xhtml

meaningful difference in either direction, implying the treatments have similar effects, the results were categorised as true negative. If the 95% confidence interval included a meaningful difference in either direction (i.e. trial failed to answer the primary question), the results were categorised as being truly inconclusive.

Where a single primary outcome and primary time point were not explicitly identified we utilised the following hierarchy to determine which primary outcome would be used in the analysis:

- Explicitly defined primary outcome
- Outcome used in power calculation
- Main outcome stated in trial objectives
- First outcome reported in sample size calculation

If a primary time point was not reported we used the first follow up time point as this is when we would expect the intervention to have had the greatest effect.

Our preliminary analysis of the qualitative documentary data involved the reading and re-reading of source documents and the extracted descriptions to consolidate our understanding of the development work undertaken in each study. Using a reflective and iterative process we undertook thematic analysis to distil, structure and make sense of intervention development activity by coding and organising data into themes and subthemes. Each theme and sub-theme provided a coherent description of the development work undertaken for each study, which were then synthesised into short descriptors to allow us to produce summary tables. The summary tables comprised a row for each study with columns for each theme and, where relevant, each subtheme. A second researcher checked, discussed and refined descriptors to ensure accuracy. From these descriptions we then developed descriptive ratings on the quality of the intervention development. Depending on the nature of the data, ratings were categorised and the iterative process involved two researchers refining and checking ratings to ensure they reflected the summary data from each study. Table 1 also provides examples of summary data underpinning each rating. In order to provide a visual representation of the quality of intervention development work these ratings were then converted to a quality coding to indicate high quality, some or unclear quality or limited quality.

To examine the relationship between intervention development and treatment success, we applied mixed methods analytical techniques in novel ways. For each study, we combined ratings derived from the qualitative data on intervention development with the quantitative data on treatment outcomes in a joint display.

RESULTS

We included 15 studies (Figure 1), with a combined sample size of 9035 participants, 7834 of whom provided primary outcomes data. Five primary outcomes were symptom-based or clinical outcomes, seven were functional measures, two were combined measures and one assessed quality of life. Primary time points varied from immediately post-intervention to one year (median 6 months). All but one of the trials were individually randomised. Thirteen of the studies utilised a two-arm, parallel RCT design, one used a four-arm factorial design of which only two arms related to physical

For peer review only - http://bmjopen.bmj.com/site/about/guidelines.xhtml

rehabilitation, and one was a two-arm cluster RCT. The study populations were: Stroke (n=4), Neurological conditions (n=2), Inflammatory/Immune system disorders (n=2) and one each of Respiratory, Musculoskeletal, Cardiovascular, Mental Health, Accident/injuries, Renal/urogenital and other. Nine interventions were of physiotherapy, two occupational therapy, one speech and language therapy, one of multiple professions both delivering the intervention and two where different professions delivered the intervention, for example, a physiotherapist or an occupational therapist. The Chief Investigators leading the studies were physicians (n=7), physiotherapists (n=5), occupational therapists (n=1), psychologists (n=1) and methodologists (n=1). The total amount of research funding awarded was £11,361,182.

One third of studies (5/15) reported a definitive finding in favour of one of the treatment arms - four studies in favour of the new treatment, one in favour of the control. Of those with negative results, eight studies reported a true negative (no difference) outcome, one was inconclusive in favour of the new treatment, and one inconclusive in favour of the control treatment (Figures 2 and 3).

Qualitative data informed two themes and ten sub-themes which enabled us to develop data-driven quality ratings (Table 1):

1. *Preparatory work* (Need for the study, underpinning theory for the intervention, co-design, context considerations and intervention piloting)
2. *Intervention and control* (Intervention content and dose, individual tailoring, adherence strategies, standardised training, control content and dose)

Table 2 presents the integrative analysis using a joint display. No single study was deemed to be high quality in each sub-theme. The best rated studies did not achieved the highest quality rating in only one area – co-design. These studies reported only expert clinical input into co-designing the intervention with a lack of clear patient and public involvement, however, two of them reported a definitive trial outcome in favour of the new intervention. There does not appear to be a single aspect of intervention development driving study outcomes. This said, those with lower quality development work appear more likely to show no difference in outcomes compared with those with higher quality development work. Some areas of intervention development appear to be improving with time, these being articulating a clear need and theoretical underpinning, co-design, piloting and descriptions of intervention and control components.

For peer review only

For peer review only - http://bmjopen.bmj.com/site/about/guidelines.xhtml

Table 1 Description of themes, subthemes and quality ratings with examples

Theme	Sub-theme	Description of rating	Examples of data supporting rating	Rating
**Preparatory work**	**Need for the study**	Multiple sources of evidence of need for the study e.g. recent systematic review, guidelines, high level reports, commissioned research, national audit	International task force highlighted lack of evidence and need for evaluation. Cochrane review drew similar conclusions.	▲
		Single source of evidence / non-systematic review to support need for study	Old systematic review indicates paucity of high quality research.	■
		Lack of clarity or underpinning evidence regarding need for study	Poor justification for the study. Evidence cited doesn't support the need for this particular study.	●
	**Theoretical underpinning**	Theoretical underpinning described	Physiological and psychological theories underpinning the intervention described in detail.	▲
		Lacks clear theoretical underpinning	No information provided regarding the theoretical basis for the intervention provided.	■
	**Co-design**	Good PPI and expert clinical input	Patients and clinicians helped develop the intervention.	▲
		Good PPI but weak or no expert clinical input / Good clinical input but unclear or no PPI	Clinicians contributed to the intervention development but no indication of service user involvement.	■
		No co-design	No co-design was undertaken to develop the intervention.	●
	**Contextual considerations**	Context considered	The use of different professionals in delivering the intervention reflected the real world situation of how this would occur in practice.	▲
		Context not adequately considered	There was a lack of understanding of relevant context and factors needed for intervention development and delivery.	●
	**Piloting of intervention**	Pilot conducted, evaluated and findings addressed for main evaluation	The pilot data helped refine the intervention for evaluation in the main trial.	▲
		Pilot conducted but findings not clearly addressed in intervention for main evaluation	The pilot work led to a modification of the control intervention but unclear as to whether this also happened for the novel intervention.	■
		No pilot reported	No piloting of intervention reported	●

For peer review only - http://bmjopen.bmj.com/site/about/guidelines.xhtml

**Intervention and control**	**Content and dose**	Intervention components and dose clearly described	The content and the dose of the exercise programme was described in detail.	▲
		Intervention components clearly described but dose was not standardised	The content of the programme was well described but no specific dose was prescribed.	■
		Intervention not replicable from description of components and dose	Intervention was based on usual practice and had no protocol or guidance on minimum dose.	●
	**Tailoring**	Formalised assessment to inform tailoring	An assessment tool was used to determine the individuals level of exercise intensity	▲
		Clinical judgement only used to inform tailoring	Therapists used their clinical judgement to individually tailor programmes.	■
		Not adequately reported	Intervention individually tailored but no information as to how this was undertaken.	●
	**Adherence support strategies**	Explicit strategies to support adherence to the intervention clearly reported	Specific adherence strategies described as part of the intervention.	▲
		No clear information regarding adherence support strategies	No information reported regarding adherence strategies.	●
		Supporting adherence is not relevant to the intervention	The intervention was passive and adherence strategies not relevant.	NA
	**Intervention training**	Standardised training in intervention received +/- additional/ongoing support or training	Staff attended a 1.5 day training session and had an additional support session with ongoing contact from research team.	▲
		No standardised intervention training received but staff delivering described to be experienced in the intervention or training of staff unclear/not reported	Staff have post graduate training in the intervention but no study specific training reported.	■
	**Control description**	Active control/attention control/usual care with some standardised components	Control was an active intervention that differed from intervention only in terms of delivery setting.	▲
		Usual care had no standardised components	Control was usual care and was not standardised between sites.	■

Key: ▲ High quality ■ Some/Unclear quality ● Limited quality

For peer review only

For peer review only - http://bmjopen.bmj.com/site/about/guidelines.xhtml

Table 2 Joint display of the quality of intervention development work of NIHR funded trial of rehabilitation and treatment success

Year of Publication	Need	Theory	Co-design	Context	Pilot	Intervention Content	Tailored	Adherence strategies	Training delivery	Control description	Treatment success
2012	High	High	Some/Unclear	High	High	High	High	High	High	High	Statistically significant in favour of intervention
2015	High	High	Some/Unclear	High	High	High	High	High	High	High	Statistically significant in favour of intervention
2013	High	High	Some/Unclear	High	High	High	High	High	High	High	True negative (No difference)
2011	High	High	High	High	Limited	High	High	High	High	High	True negative (No difference)
2014	High	High	High	High	Some/Unclear	Some/Unclear	High	High	High	High	True negative (No difference)
2012	High	High	Some/Unclear	High	High	Some/Unclear	High	High	Some/Unclear	High	Inconclusive in favour of intervention
2015	High	High	Limited	High	Some/Unclear	High	High	NA	High	Some/Unclear	Statistically significant in favour of control
2016	High	High	Some/Unclear	High	Some/Unclear	Limited	High	High	Some/Unclear	Some/Unclear	True negative (No difference)
2007	Limited	High	Limited	High	Limited	High	Some/Unclear	High	High	Some/Unclear	True negative (No difference)
2004	Some/Unclear	Some/Unclear	Limited	High	Limited	High	High	Some/Unclear	Some/Unclear	High	Statistically significant in favour of intervention
2010	High	Some/Unclear	Limited	Limited	Limited	High	Some/Unclear	High	Some/Unclear	High	True negative (No difference)
2005	Some/Unclear	High	Some/Unclear	Limited	Some/Unclear	High	Some/Unclear	Some/Unclear	Some/Unclear	High	Inconclusive in favour of control
2016	Limited	Some/Unclear	Some/Unclear	High	Limited	Limited	High	Some/Unclear	Some/Unclear	Some/Unclear	True negative (No difference)
2004	Some/Unclear	Some/Unclear	Limited	High	Limited	Limited	Limited	NA	Some/Unclear	Some/Unclear	Statistically significant in favour of intervention
2007	Limited	High	Limited	Limited	Limited	Limited	Limited	Some/Unclear	Some/Unclear	Some/Unclear	True negative (No difference)

Key:

High quality (green triangle) Some/Unclear quality (amber square) Limited quality (red circle)

For peer review only

For peer review only - http://bmjopen.bmj.com/site/about/guidelines.xhtml

DISCUSSION

We found that only one third (5/15) of the randomised controlled trials of rehabilitation funded by the NIHR HTA programme successfully demonstrated a statistically significant effect for one of the randomised groups in each trial. Four (27%) trials found an effect in favour of the 'new' intervention. We were able to use contemporary research methods to develop an assessment of the quality of development work and assessed the included trials to be of varied quality in terms of intervention development work. In general, we found that those studies with better quality intervention development work were more likely to report treatment success, although older, possibly less well reported studies also reported effective interventions. We found that interventions that were less well developed were more likely to lead to results categorised as truly negative, i.e. which excluded a meaningful difference in outcome in either direction. Developments in complex intervention evaluation[5], reporting standards[14 15] and involving patients and the public in research[16] have occurred since the inception of the HTA programme and as such some development work may not have been reported in the included studies. There are of course other factors that influence trial findings, including trial conduct, however our question was specifically determined to explore what if any relationship existed between intervention development and outcomes and not in the effectiveness of particular interventions.

A strength of our study is the use of integrative mixed methods analysis which has enabled us to explore the relationship between development work and outcome. This rarely used approach in evidence synthesis[11] has given us a unique insight that would not have been possible using a quantitative or qualitative analysis alone. A limitation of our work could be the focus on a single UK funding stream which does not necessarily reflect the body of research funded from other sources and therefore the quality of intervention development work is not necessarily generalizable. However, the fact the NIHR HTA programme is the single largest funder of randomised controlled trials of applied health research in the UK that published detailed monographs of their funded studies, several of which were over 200 pages in length, along with supporting publications provided a detailed and rich source of data beyond what would normally be available in journal-based peer reviewed publications alone. We were able to retain the essence and nuances of the qualitative data whilst developing categorical ratings of quality to help us better explore the relationship between development work and treatment success.

Our findings are similar to those of Dent and Raftery[9] in relation to those trials showing a benefit who reported 19% (16/85) of studies found in favour of the new intervention. It has been suggested that a 50% success rate is a good investment for healthcare research,[17] however, our findings indicate that the studies we reviewed fell well below this. In contrast,, we observed a considerably larger proportion of true negative studies (8/15; 53%) compared with 19/85 (22%) reported by Dent and Raftery.[9] The difference is even greater when compared with a review of cancer trials in the USA where only 2% of trials found a true negative outcome.[10] The reasons for the differences are unclear but could include the pragmatic nature of HTA funded trials and the relative smaller effect sizes often associated with trials of rehabilitation.[18]

It has been recently suggested that RCTs should only be undertaken if they are justified both scientifically and ethically by having a clear hypothesis and established uncertainty[19] and our findings support that by way of good quality intervention development work. Our findings also align with the

For peer review only - http://bmjopen.bmj.com/site/about/guidelines.xhtml

elements suggested to be key for developing interventions and reducing research waste by increasing the likelihood of success[20] which will form a comprehensive supplement to the development phase of the MRC Framework. By increasing effort and focus on developing rehabilitation and other interventions in the future researchers and funding bodies could increase the possibility of a definitive trial reporting significant findings after much investment of time and money.

CONCLUSIONS

Despite much research effort and funding, only four out of fifteen evaluations of 'new' rehabilitation interventions funded by the NIHR HTA programme were found to be unequivocally effective. Most studies reported no difference in outcome between study arms. We have used mixed methods research to explore the relationship between intervention development work and treatment success and developed a method of assessing the quality of this work which suggests comprehensive intervention development work may have a positive relationship with treatment success.

RECOMMENDATIONS

As this was an exploratory study, further work should be undertaken to establish the validity of quality assessment of intervention development work. This said, researchers and funding agencies should not undervalue the potential benefit of high quality intervention development work prior to definitive randomised controlled trials to reduce the likelihood of a null outcome and improve current rates of treatment success.

ACKNOWLEDGEMENT

This work was supported by the NIHR Collaboration for Leadership in Applied Health Research and Care South West Peninsula (PenCLAHRC). The views expressed are those of the authors and not necessarily the NHS, the NIHR or the Department of Health and Social Care.

COMPETING INTERESTS

None

AUTHOR CONTRIBUTION

VG: Conception and design, data collection, analysis and interpretation, drafting and approving the manuscript;

JH: Design, data collection, analysis and interpretation, drafting and approving the manuscript;

JF: Data collection, analysis, revising and approving the manuscript;

KF: Data collection, revising and approving the manuscript;

CP: Data collection, revising and approving the manuscript;

DR: Conception, revising and approving the manuscript.

For peer review only - http://bmjopen.bmj.com/site/about/guidelines.xhtml

REFERENCES

1. World Health Organization. Rehabilitation in health systems. Geneva, 2017.
2. De Souza L. Theories about therapies are underdeveloped. *Physiotherapy Research International* 1998;3(3):iv-vi.
3. Hislop HJ. Tenth Mary McMillan lecture. The not-so-impossible dream. *Phys Ther* 1975;55(10):1069-80. [published Online First: 1975/10/01]
4. Mayo N, Kaur N, Barbic S, et al. How have research questions and methods used in clinical trials published in Clinical Rehabilitation changed over the last 30 years? *Clinical Rehabilitation* 2016;30(9):847-64.
5. Medical Research C. A framework for development and evaluation of RCT's for complex interventions to improve health. London: Medical Research Council, 2000.
6. Medical Research Council. Developing and evaluating complex interventions: new guidance. London: Medical Research Council, 2008.
7. Richards D. The complex interventions framework. In: Richards D, Hallberg I, eds. Complex interventions in health: an overview of research methods. Abingdon: Routledge 2015:5.
8. Chalmers I, Glasziou P. Avoidable waste in the production and reporting of research evidence. *Lancet* 2009;374(9683):86-9. doi: 10.1016/S0140-6736(09)60329-9 [published Online First: 2009/06/16]
9. Dent L, Raftery J. Treatment success in pragmatic randomised controlled trials: a review of trials funded by the UK Health Technology Assessment programme. *Trials* 2011;12:109. doi: 10.1186/1745-6215-12-109 [published Online First: 2011/05/06]
10. Djulbegovic B, Kumar A, Soares HP, et al. Treatment success in cancer: new cancer treatment successes identified in phase 3 randomized controlled trials conducted by the National Cancer Institute-sponsored cooperative oncology groups, 1955 to 2006. *Arch Intern Med* 2008;168(6):632-42. doi: 10.1001/archinte.168.6.632 [published Online First: 2008/03/26]
11. Petticrew M, Rehfuess E, Noyes J, et al. Synthesizing evidence on complex interventions: how meta-analytical, qualitative, and mixed-method approaches can contribute. *J Clin Epidemiol* 2013;66(11):1230-43. doi: 10.1016/j.jclinepi.2013.06.005 [published Online First: 2013/08/21]
12. Barbour RS. The case for combining qualitative and quantitative approaches in health services research. *J Health Serv Res Policy* 1999;4(1):39-43. doi: 10.1177/135581969900400110 [published Online First: 1999/05/27]
13. Mohler R, Kopke S, Meyer G. Criteria for Reporting the Development and Evaluation of Complex Interventions in healthcare: revised guideline (CReDECI 2). *Trials* 2015;16:204. doi: 10.1186/s13063-015-0709-y [published Online First: 2015/05/04]
14. Hoffmann TC, Glasziou PP, Boutron I, et al. Better reporting of interventions: template for intervention description and replication (TIDieR) checklist and guide. *BMJ* 2014;348:g1687. doi: 10.1136/bmj.g1687 [published Online First: 2014/03/13]
15. Moher D, Schulz KF, Altman DG. The CONSORT statement: revised recommendations for improving the quality of reports of parallel group randomised trials. *Lancet* 2001;357:1191-94.
16. Boote J, Baird W, Sutton A. Public involvement in the systematic review process in health and social care: a narrative review of case examples. *Health Policy* 2011;102(2-3):105-16. doi: 10.1016/j.healthpol.2011.05.002 [published Online First: 2011/06/07]
17. Djulbegovic B. Acknowledgment of uncertainty: a fundamental means to ensure scientific and ethical validity in clinical research. *Curr Oncol Rep* 2001;3(5):389-95. [published Online First: 2001/08/08]
18. Angst F, Aeschlimann A, Stucki G. Smallest detectable and minimal clinically important differences of rehabilitation intervention with their implications for required sample sizes using WOMAC and SF-36 quality of life measurement instruments in patients with osteoarthritis of the lower extremities. *Arthritis and rheumatism* 2001;45(4):384-91. doi:

For peer review only - http://bmjopen.bmj.com/site/about/guidelines.xhtml

10.1002/1529-0131(200108)45:4<384::AID-ART352>3.0.CO;2-0 [published Online First: 2001/08/15]

19. De Meulemeester J, Fedyk M, Jurkovic L, et al. Many randomized clinical trials may not be justified: a cross-sectional analysis of the ethics and science of randomized clinical trials. *J Clin Epidemiol* 2018;97:20-25. doi: 10.1016/j.jclinepi.2017.12.027 [published Online First: 2018/01/07]

20. Bleijenberg N, de Man-van Ginkel JM, Trappenburg JCA, et al. Increasing value and reducing waste by optimizing the development of complex interventions: Enriching the development phase of the Medical Research Council (MRC) Framework. *International journal of nursing studies* 2018;79:86-93. doi: 10.1016/j.ijnurstu.2017.12.001 [published Online First: 2017/12/09]

For peer review only

For peer review only - http://bmjopen.bmj.com/site/about/guidelines.xhtml

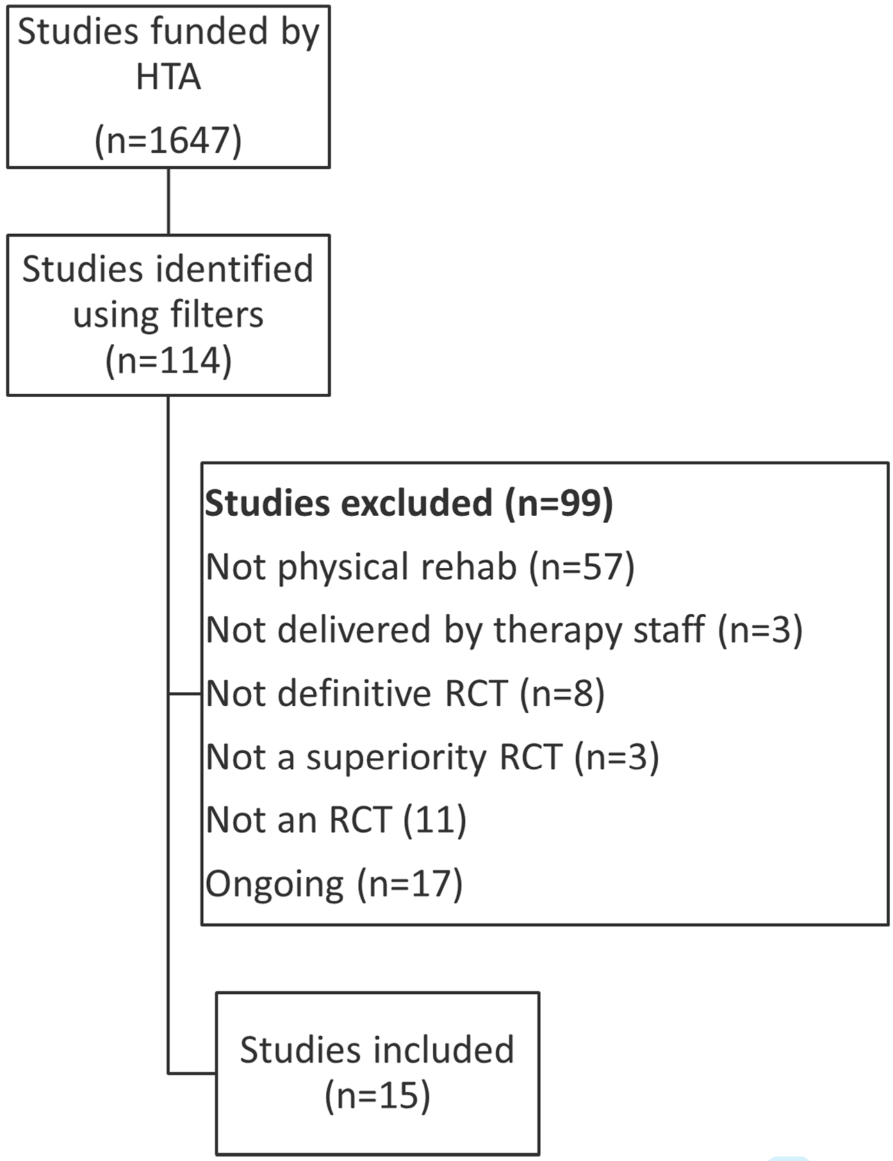

Figure 1 Study selection

only

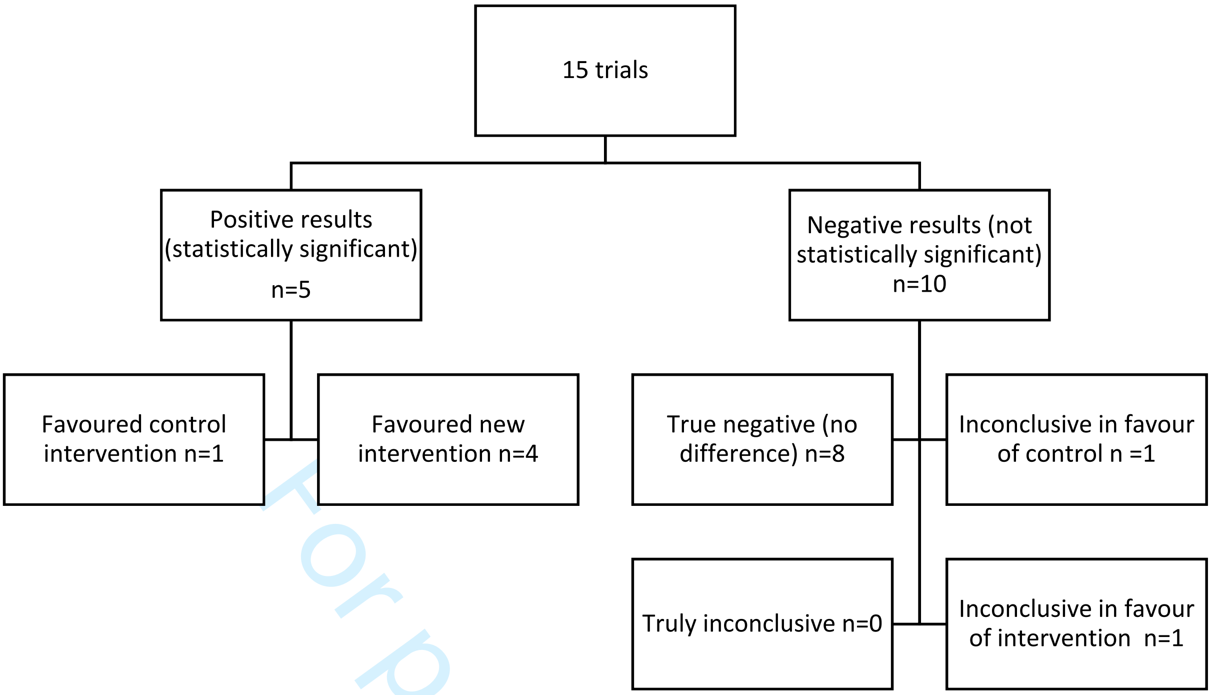

Figure 1 Classification of primary outcome

For peer review only

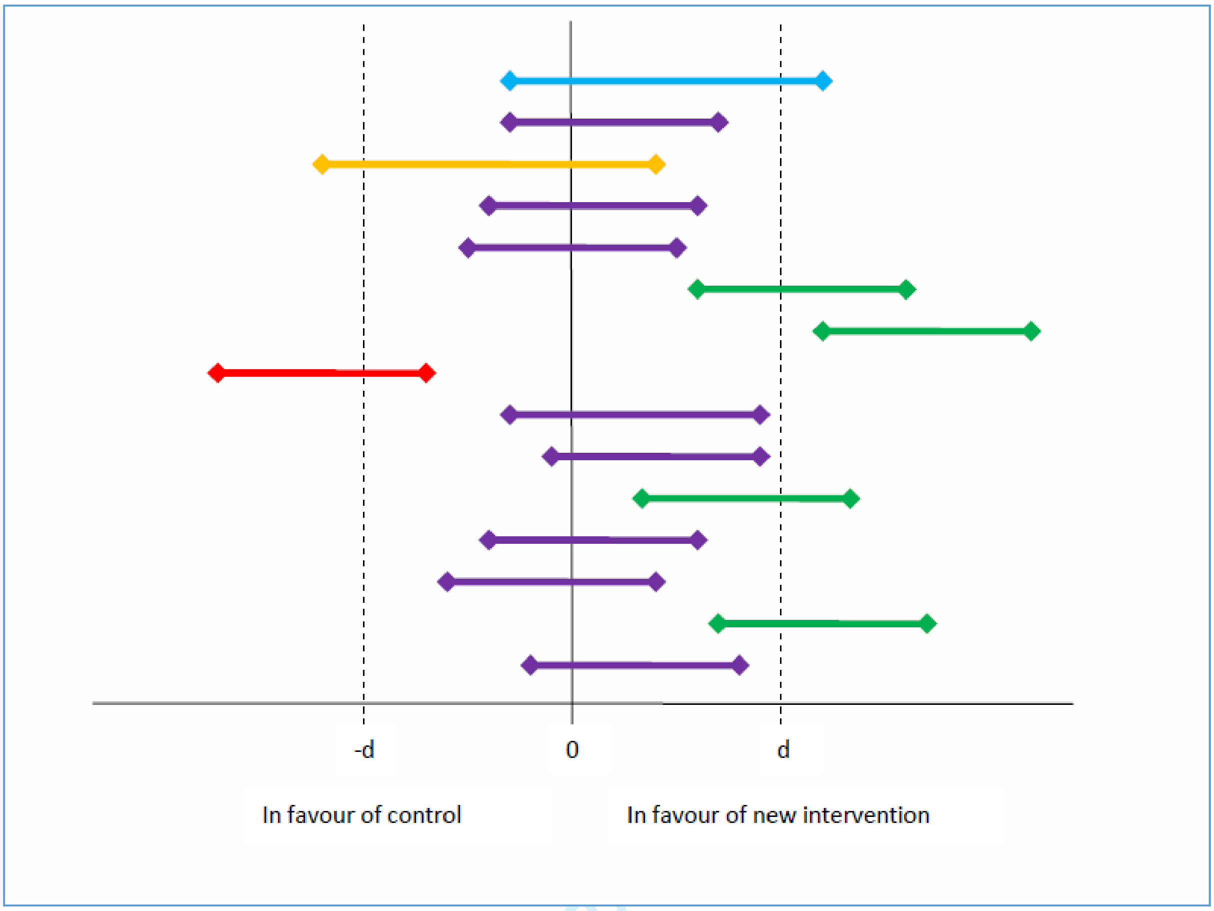

Figure 1 Treatment success of included trials based on 95% Confidence Interval and Minimal Clinically Important Difference from trial sample size calculation (d)

Key: *Green = statistically significantly in favour of intervention; Red = statistically significantly in favour of control; Blue = Inconclusive in favour of intervention; Yellow = Inconclusive in favour of control; Purple = True negative (no difference)*

For peer review only - http://bmjopen.bmj.com/site/about/guidelines.xhtml

BMJ Open

Intervention development and treatment success in UK Health Technology Assessment funded trials of rehabilitation: a mixed methods analysis

Journal:	*BMJ Open*
Manuscript ID	bmjopen-2018-026289.R1
Article Type:	Research
Date Submitted by the Author:	22-Apr-2019
Complete List of Authors:	Goodwin, Victoria; University of Exeter Medical School<br>Hill, Jacqueline; University of Exeter Medical School<br>Fullam, James; University of Exeter Medical School<br>Finning, Katie; University of Exeter Medical School<br>Pentecost, C; University of Exeter Medical School<br>Richards, David; University of Exeter, Medical School
**Primary Subject Heading**:	Health services research
Secondary Subject Heading:	Rehabilitation medicine
Keywords:	rehabilitation, intervention development, mixed methods, randomised controlled trials

SCHOLARONE™
Manuscripts

Intervention development and treatment success in UK Health Technology Assessment funded trials of rehabilitation: a mixed methods analysis

*Victoria A Goodwin, University of Exeter, Exeter UK

Jacqueline J Hill, University of Exeter, Exeter UK

James Fullam, University of Exeter, Exeter UK

Katie Finning, University of Exeter, Exeter UK

Claire Pentecost, University of Exeter, Exeter UK

David A Richards, University of Exeter, Exeter UK

* Corresponding author: 2.26 South Cloisters, University of Exeter Medical School, St Luke's Campus, Magdalen Road, Exeter EX1 2LU. Telephone 01392 722745 v.goodwin@exeter.ac.uk

For peer review only

Keywords

Rehabilitation, randomised controlled trials, quality, intervention development, mixed methods

For peer review only - http://bmjopen.bmj.com/site/about/guidelines.xhtml

Abstract

Objectives: Physical rehabilitation is a complex process and trials of rehabilitation interventions are increasing in number but often report null results. This study aimed to establish treatment success rates in physical rehabilitation trials funded by the National Institute of Health Research Health Technology Assessment (NIHR HTA) programme and examine any relationship between treatment success and the quality of intervention development work undertaken.

Design: Mixed methods study

Setting: UK

Methods:

The NIHR HTA portfolio was searched for all completed definitive randomised controlled trials of physical rehabilitation interventions from inception to July 2016. Treatment success was categorised according to criteria developed by Djulbegovic and colleagues. Detailed textual data regarding any intervention development work were extracted from trial reports and supporting publications and informed the development of quality ratings. Mixed methods integrative analysis was undertaken to explore the relationship between quantitative and qualitative data using joint displays.

Results: Fifteen trials were included in the review. Five reported a definitive finding, four of which were in favour of the 'new' intervention. Eight trials reported a true negative (no difference) outcome. Integrative analysis indicated those with lower quality intervention development work were less likely to report treatment success.

Conclusions: Despite much effort and funding, most physical rehabilitation trials report equivocal findings. Greater focus on high quality intervention development may reduce the likelihood of a null result in the definitive trial, alongside high quality trial methods and conduct.

Strengths and limitations of this study:

- To our knowledge, this study is the first to use mixed methods integrative analyses to explore the relationship between quality of intervention development work and treatment success.
- Using the NIHR HTA Journal monographs, published protocols and other supporting publications for each study together provided a detailed and rich source of data beyond what would be found in a single traditional journal publication.
- The study reviewed randomised controlled trials of physical rehabilitation from a single UK funder as an exemplar and therefore findings may not be representative of other complex interventions or other funding bodies.

For peer review only

BACKGROUND

Rehabilitation is *"a set of interventions designed to optimise function and reduce disability in individuals with health conditions in interaction with their environment".*[1] and is an essential aspect of healthcare provision. By its very nature rehabilitation in clinical practice is an individually focused, complex activity, involving interventions that are multi-faceted and often implicit in nature[2] and as such, historically, this has been viewed as a barrier to undertaking research.[3] This said, there is a growing body of randomised controlled trials (RCTs) of rehabilitation, suggesting that these challenges can be overcome.[4] This may, in part, be supported by the publication of the Medical Research Council (MRC) Framework for developing and evaluating complex interventions.[5 6]

The MRC framework was developed to optimise the likelihood that new interventions are not rejected as being ineffective when inadequate effort has been made in the development of the intervention.[7] Likewise, Chalmers and Glasziou[8] highlighted the importance of avoiding research waste and recommended that sufficient effort is made to ensure the relevant research questions are identified and addressed using high quality research methods. However, there appears to have been no formal evaluation of the impact of using the development component of the framework on trial outcomes and whether we are observing evidence of effective interventions being developed.

Previous UK[9] and USA[10] reviews synthesised successful and non-successful treatment outcomes from trials of new interventions in order to assess the equipoise principle and to understand what return has been achieved on the investment made by those taking part in the trials, researchers and funders. Dent and Raftery[9] reported 24% (20/85) primary outcome comparisons as having a positive result, of which 16/85 (19%) were in favour of the new intervention, with 19/85 (22%) comparisons reporting a true negative outcome. However, these authors did not focus on rehabilitation interventions, nor did they seek to understand factors that may impact on treatment success, such as the quality or intensity of intervention development pre-trial procedures. Informal discussions with colleagues in the UK and internationally noted that an increasing number of publically funded, large RCTs evaluating physical rehabilitation interventions had reported null findings. Similar concerns have been reported in studies of public health interventions.[11 12] Our study, therefore, sought to assess this observation and also explore whether intervention development activities contributed to treatment success using the National Institute of Health Research Health Technology Assessment programme (NIHR HTA) as an exemplar.

We aimed to use data from the NIHR HTA to:

(a) Establish the treatment outcomes of funded RCTs of physical rehabilitation;

(b) Establish how many new interventions were found to be effective;

(c) Examine what work had been done in terms of developing the new intervention;

(d) Examine the relationship between (a) and (c).

We adopted a mixed methods approach to address the study aims. Although evidence of using integrative mixed methods approaches in synthesising evidence on complex interventions is limited, mixing together qualitative and quantitative data can generate understanding that has the potential to be greater than the sum of the individual parts.[13]

For peer review only

METHODS

Design

We undertook a review of NIHR HTA funded randomised controlled trials of physical rehabilitation interventions using narrative synthesis of outcomes and mixed methods analysis of the relationship between intervention development and categorical treatment outcomes using joint displays.

Patient and Public Involvement

Patients and the public were not involved in this study.

Data sources and inclusion criteria

We included superiority randomised controlled trials of physical rehabilitation funded by the NIHR HTA programme. The interventions could be delivered by a single profession or be multi-professional. The NIHR HTA programme is the leading public funding source for randomised controlled trials (RCTs) in the UK and trials of rehabilitation are increasingly part of the portfolio. We only included completed RCTs whose main trial findings were reported in an HTA monograph or peer-reviewed publication in order to establish treatment success. We excluded: pilot and feasibility RCTs as they do not aim to assess the efficacy or effectiveness of interventions;[14] studies where the interventions were primarily psychological or cognitive as the focus of the study was physical rehabilitation; where there was a lack of a clear primary outcome (including primary time point) or where the primary outcome findings were not reported with a 95% confidence interval (CI) as these data were required to assess treatment success.

Search and screening

We searched the HTA Project Portfolio (since superseded by the NIHR Journals Library https://www.journalslibrary.nihr.ac.uk/#/) from inception to July 2016 using the following keywords: physiotherap*OR occupational therap* OR speech and language therap* OR rehabilitation. We removed duplicates and then titles and scientific abstracts were reviewed for potential inclusion by one person and checked by a second. Subsequently full text reports were screened for inclusion by one person and checked by a second. Any disagreements were discussed and agreed with a third person.

Data extraction

All data were extracted by one person and checked by a second. Discrepancies were discussed and resolved with a third person.

Quantitative Trial data: Data extracted from each trial publication included trial design, target population, health categories (using the Health Research Classification System), primary outcome(s) and time point, minimal important clinical difference (MCID) that the trial aimed to detect, planned and achieved sample size, and primary outcome results with 95% CI. We also recorded the professional background of the Chief Investigator and amount of funding awarded.

Qualitative Intervention development data: Using the revised version of Criteria for Reporting the Development and Evaluation of Complex Interventions (CReDECI 2)[15] and the Template for

For peer review only - http://bmjopen.bmj.com/site/about/guidelines.xhtml

Intervention Description and Replication checklist (TIDieR)[16] as frameworks we extracted all available documentary (qualitative) data from the body of the text regarding intervention development, including descriptions of underlying theory, intervention components and reasons for selection, intended interactions between components, contextual considerations, piloting of intervention and impact of definitive intervention to be evaluated, control components, planned intervention delivery and materials. Where additional supporting publications were cited, such as a protocol or intervention development studies, we used these as additional sources of documentary data.

Data analysis

We used summary statistics to describe the characteristics of the included studies. We categorised primary outcome findings into one of six treatment outcome categories as described by Djulbegovic and colleagues,[10] these being: 1) statistically significant in favour of the new treatment, 2) statistically significant in favour of the control treatment 3) true negative, 4) truly inconclusive, 5) inconclusive in favour of new treatment or 6) inconclusive in favour of the control treatment. This was achieved by comparing the 95% confidence interval for the difference in primary outcome to the difference specified in the sample size calculation.[9] If the 95% confidence interval excluded a meaningful difference in either direction, implying the treatments have similar effects, the results were categorised as true negative. If the 95% confidence interval included a meaningful difference in either direction (i.e. trial failed to answer the primary question), the results were categorised as being truly inconclusive.

Where a single primary outcome and primary time point were not explicitly identified we utilised the following hierarchy to determine which primary outcome would be used in the analysis:

- Explicitly defined primary outcome
- Outcome used in power calculation
- Main outcome stated in trial objectives
- First outcome reported in sample size calculation

If a primary time point was not reported we used the first follow up time point as this is when we would expect the intervention to have had the greatest effect.

Our preliminary analysis of the qualitative documentary data involved the reading and re-reading of source documents and the extracted descriptions to consolidate our understanding of the development work undertaken in each study. Using a reflective and iterative process we undertook thematic analysis to distil, structure and make sense of intervention development activity by coding and organising data into themes and subthemes. Each theme and sub-theme provided a coherent description of the development work undertaken for each study, which were then synthesised into short descriptors to allow us to produce summary tables. The summary tables comprised a row for each study with columns for each theme and, where relevant, each subtheme. A second researcher checked, discussed and refined descriptors to ensure accuracy. From these descriptions we then developed descriptive ratings on the quality of the intervention development. Depending on the nature of the data, ratings were categorised and the iterative process involved two researchers refining and checking ratings to ensure they reflected the summary data from each study. In order to provide a visual representation of the quality of intervention development work these ratings were then converted to a quality coding to indicate high quality, some or unclear quality or limited quality.

For peer review only - http://bmjopen.bmj.com/site/about/guidelines.xhtml

To examine the relationship between intervention development and treatment success, we applied mixed methods analytical techniques in novel ways. For each study, we combined ratings derived from the qualitative data on intervention development with the quantitative data on treatment outcomes in a joint display.

RESULTS

We included fifteen RCTs (Figure 1),[17-31] of which thirteen used a two-arm, parallel RCT design, one was a two-arm cluster RCT and one was a four-arm factorial design (of which only two arms related to physical rehabilitation). Table 1 provides a summary of the population, intervention, control and outcomes for each study. The combined sample size was 9035 participants, 7834 of whom provided primary outcome data. Five primary outcomes were symptom-based or clinical outcomes, seven were functional measures, two were combined measures and one assessed quality of life. Primary time points varied from immediately post-intervention to one year (median 6 months). The health categories were: Stroke (n=4), Neurological conditions (n=2), Inflammatory/Immune system disorders (n=2), Respiratory (n=1), Musculoskeletal (n=1), Cardiovascular (n=1), Mental Health (n=1), Accident/injuries (n=1), Renal/urogenital (n=1) and other (n=1). Seven interventions were delivered by physiotherapists, one by occupational therapists, one by speech and language therapists, one by nurses, two could be delivered by either a physiotherapist or a nurse, two could be delivered by a physiotherapist or an occupational therapist and one was delivered by both a physiotherapist and an occupational therapist. The Chief Investigators leading the studies were physicians (n=7), physiotherapists (n=5), occupational therapists (n=1), psychologists (n=1) and methodologists (n=1). The total amount of research funding awarded was £11,361,182.

One third of studies (5/15) reported a definitive finding in favour of one of the treatment arms - four studies in favour of the new treatment, one in favour of the control. Of those with negative results, eight studies reported a true negative (no difference) outcome, one was inconclusive in favour of the new treatment, and one inconclusive in favour of the control treatment (Figures 2 and 3).

Qualitative data informed two themes and ten sub-themes which enabled us to develop data-driven quality ratings:

1. *Preparatory work* (Need for the study, underpinning theory for the intervention, co-design, context considerations and intervention piloting)
2. *Intervention and control* (Intervention content and dose, individual tailoring, adherence strategies, standardised training, control content and dose)

Table 2 provides examples of summary data underpinning each rating. Table 3 presents the integrative qualitative and quantitative analysis using a joint display. No single study was deemed to be high quality in each sub-theme. This said, the two best rated studies reported only expert clinical input into co-designing the intervention with a lack of clear patient and public involvement, however, they reported a definitive trial outcome in favour of the new intervention. There does not appear to be a single aspect of intervention development driving study outcomes. This said, those with lower quality development work appear more likely to show no difference in outcomes compared with those with higher quality development work. Some areas of intervention development appear to be improving

For peer review only - http://bmjopen.bmj.com/site/about/guidelines.xhtml

with time, these being articulating a clear need and theoretical underpinning, co-design, piloting and descriptions of intervention and control components.

For peer review only

For peer review only - http://bmjopen.bmj.com/site/about/guidelines.xhtml

Table 1 Summary of included studies

Author (year published)	Population (sample size)	Intervention	Control	Primary Outcome
McCarthy et al (2004)	People with knee osteoarthritis (n=225)	Twice weekly exercise group for 8 weeks plus home exercises	Home exercises	Aggregate Locomotor Function score
Vickers et al (2004)	People with chronic headache (n=401)	Up to 12 acupuncture treatments plus usual care	Usual care from General Practitioner	Weekly headache score
Epps et al (2005)	Children with juvenile arthritis (n=101)	8 hydrotherapy and 8 land based sessions over 2 weeks followed by weekly/fortnightly hydrotherapy for 2 months	16 land based exercise sessions over 2 weeks followed by weekly or fortnightly land based exercise sessions	Disease status calculated from Childhood Health Assessment Questionnaire (CHAQ), physicians' global assessment of disease activity, parents' global assessment of overall well-being, number of joints with limited ROM, number of active joints, erythrocyte sedimentation rate.
Weindling et al (2007)	Children with cerebral palsy (n=88)	Regular physiotherapy (usual care) plus additional weekly session from physiotherapy assistant for 6 months	Usual care (regular physiotherapy)	Gross Motor Function Measure
Jolly et al (2007)	People with myocardial infarction or revascularisation (n=525)	Home-based self-help manual plus up to 3 face to face and 1 phone call support over 12 weeks	Centre-based cardiac rehabilitation	Multiple primary outcomes (Incremental shuttle walk test; Hospital anxiety and depression scale; smoking; blood pressure; serum cholesterol)
Lawson et al (2010)	People with Chronic Obstructive Pulmonary Disease (n=326)	Twice weekly community-based pulmonary rehabilitation	Twice weekly hospital-based pulmonary rehabilitation	Endurance Shuttle Walk Test
Glazener et al (2011)	Men with incontinence post-prostate surgery (n=853)	Assessment and treatment and exercise over 4 face to face sessions plus advice leaflet	Advice leaflet	Self-reported urinary incontinence
Bowen et al (2012)	Adults with aphasia or dysarthria after stroke (n=170)	Speech and language therapy visits up to 3 sessions per week for up to 16 weeks	Volunteer visits up to 3 sessions per week for up to 16 weeks	Therapy Outcome measure
Lamb et al (2012)	People with whiplash with persistent symptoms (n=599)	6 sessions of assessment and treatment/exercise over 8 weeks	Single session of advice	Neck Disability Index
Underwood et al (2013)	Care home residents (n=781)	Twice weekly exercise group for a year	Depression awareness training for care home staff	Geriatric Depression Scale
Logan et al (2014)	People with stroke (n=568)	Up to 12 therapy visits to increase outdoor mobility plus verbal/written advice	Verbal/written advice	SF-36 Social function domain
Williams et al (2015)	People with rheumatoid arthritis (n=490)	6 sessions of exercise plus home exercises over 12 weeks	Single assessment advice session with 2 further optional sessions over 12 weeks (no exercises)	Michigan Hand Outcome Questionnaire
Langhorne et al (2015)	People with stroke (n=2104)	3 additional out of bed sessions per day for up to 2 weeks	Usual care	Modified Rankin Scale
Sackley et al (2016)	Care home residents with stroke (n=1042)	Individualised occupational therapy	No occupational therapy	Barthel Index
Clarke et al (2016)	People with Parkinson's (n=762)	Up to 8 individualised sessions of Physiotherapy and up to 8 individualised sessions of occupational therapy	No therapy	Nottingham Extended Activities of Daily Living

For peer review only - http://bmjopen.bmj.com/site/about/guidelines.xhtml

For peer review only

Table 2 Description of themes, subthemes and quality ratings with examples

Theme	Sub-theme	Description of rating	Examples of data supporting rating	Rating
**Preparatory work**	**Need for the study**	Multiple sources of evidence of need for the study e.g. recent systematic review, guidelines, high level reports, commissioned research, national audit	International task force highlighted lack of evidence and need for evaluation. Cochrane review drew similar conclusions.	▲
		Single source of evidence / non-systematic review to support need for study	Old systematic review indicates paucity of high quality research.	■
		Lack of clarity or underpinning evidence regarding need for study	Poor justification for the study. Evidence cited doesn't support the need for this particular study.	●
	**Theoretical underpinning**	Theoretical underpinning described	Physiological and psychological theories underpinning the intervention described in detail.	▲
		Lacks clear theoretical underpinning	No information provided regarding the theoretical basis for the intervention provided.	■
	**Co-design**	Good PPI and expert clinical input	Patients and clinicians helped develop the intervention.	▲
		Good PPI but weak or no expert clinical input / Good clinical input but unclear or no PPI	Clinicians contributed to the intervention development but no indication of service user involvement.	■
		No co-design	No co-design was undertaken to develop the intervention.	●
	**Contextual considerations**	Context considered	The use of different professionals in delivering the intervention reflected the real world situation of how this would occur in practice.	▲
		Context not adequately considered	There was a lack of understanding of relevant context and factors needed for intervention development and delivery.	●
	**Piloting of intervention**	Pilot conducted, evaluated and findings addressed for main evaluation	The pilot data helped refine the intervention for evaluation in the main trial.	▲
		Pilot conducted but findings not clearly addressed in intervention for main evaluation	The pilot work led to a modification of the control intervention but unclear as to whether this also happened for the novel intervention.	■
		No pilot reported	No piloting of intervention reported	●

For peer review only

For peer review only - http://bmjopen.bmj.com/site/about/guidelines.xhtml

**Intervention and control**	**Content and dose**	Intervention components and dose clearly described	The content and the dose of the exercise programme was described in detail.	▲
		Intervention components clearly described but dose was not standardised	The content of the programme was well described but no specific dose was prescribed.	■
		Intervention not replicable from description of components and dose	Intervention was based on usual practice and had no protocol or guidance on minimum dose.	●
	**Tailoring**	Formalised assessment to inform tailoring	An assessment tool was used to determine the individuals level of exercise intensity	▲
		Clinical judgement only used to inform tailoring	Therapists used their clinical judgement to individually tailor programmes.	■
		Not adequately reported	Intervention individually tailored but no information as to how this was undertaken.	●
	**Adherence support strategies**	Explicit strategies to support adherence to the intervention clearly reported	Specific adherence strategies described as part of the intervention.	▲
		No clear information regarding adherence support strategies	No information reported regarding adherence strategies.	●
		Supporting adherence is not relevant to the intervention	The intervention was passive and adherence strategies not relevant.	NA
	**Intervention training**	Standardised training in intervention received +/- additional/ongoing support or training	Staff attended a 1.5 day training session and had an additional support session with ongoing contact from research team.	▲
		No standardised intervention training received but staff delivering described to be experienced in the intervention or training of staff unclear/not reported	Staff have post graduate training in the intervention but no study specific training reported.	■
	**Control description**	Active control/attention control/usual care with some standardised components	Control was an active intervention that differed from intervention only in terms of delivery setting.	▲
		Usual care had no standardised components	Control was usual care and was not standardised between sites.	■

Key: ▲ High quality ■ Some/Unclear quality ● Limited quality

For peer review only

Table 3 Joint display of the quality of intervention development work of NIHR funded trial of rehabilitation and treatment success

Author	Need	Theory	Co-design	Context	Pilot	Intervention Content	Tailored	Adherence strategies	Training delivery	Control description	Treatment success
Lamb[23]	▲	▲	■	▲	▲	▲	▲	▲	▲	▲	Statistically significant in favour of intervention
Williams[31]	▲	▲	■	▲	▲	▲	▲	▲	▲	▲	Statistically significant in favour of intervention
Underwood[32]	▲	▲	■	▲	▲	▲	▲	▲	▲	▲	True negative (No difference)
Glazener[20]	▲	▲	▲	▲	●	▲	▲	▲	▲	▲	True negative (No difference)
Logan[24]	▲	▲	▲	▲	■	■	▲	▲	▲	▲	True negative (No difference)
Bowen[17]	▲	▲	■	▲	▲	■	▲	▲	■	▲	Inconclusive in favour of intervention
AVERT Group[21]	▲	▲	●	▲	■	▲	▲	NA	▲	■	Statistically significant in favour of control
Sackley[26]	▲	▲	■	▲	■	●	▲	▲	■	■	True negative (No difference)
Jolly[22]	●	▲	●	▲	●	▲	■	▲	▲	■	True negative (No difference)
McCarthy[25]	■	■	●	▲	●	▲	▲	■	■	▲	Statistically significant in favour of intervention
Waterhouse[29]	▲	■	●	●	●	▲	■	▲	■	▲	True negative (No difference)
Epps[19]	■	▲	■	●	■	▲	■	■	■	▲	Inconclusive in favour of control
Clarke[18]	●	■	■	▲	●	●	▲	■	■	■	True negative (No difference)
Vickers[28]	■	■	●	▲	●	●	●	NA	■	■	Statistically significant in favour of intervention
Weindling[30]	●	▲	●	●	●	●	●	■	■	■	True negative (No difference)

Key:

▲ High quality ■ Some/Unclear quality ● Limited quality

For peer review only

DISCUSSION

We found that only one third (5/15) of the randomised controlled trials of rehabilitation funded by the NIHR HTA programme successfully demonstrated a statistically significant effect for one of the randomised groups in each trial. Four (27%) trials found an effect in favour of the 'new' intervention. Although we would not expect all studies to demonstrate effectiveness in favour of the 'new' intervention, the equipoise principle implies that there would be no difference between the proportion of studies favouring intervention or control[9]. However, this doesn't account for a null outcome. We were able to use contemporary research methods to develop an assessment of the quality of development work and assessed the included trials to be of varied quality in terms of intervention development work. In general, we found that those studies with poorer quality intervention development work were less likely to report treatment success and were more likely to lead to results categorised as truly negative, i.e. which excluded a meaningful difference in outcome in either direction. Two studies[23 31] with high quality intervention development reported treatment success although two older[25 28] and possibly less well reported trials also reported effective interventions. Developments in complex intervention evaluation[5], reporting standards[16 33] and involving patients and the public in research[34] have occurred since the inception of the HTA programme and as such some development work may have been undertaken but not reported in the older studies. A recent overview of approaches to developing interventions noted the absence of patient and public involvement[35]. In addition, there was limited evidence of piloting the intervention prior to proceeding to the full trial with only four studies reporting this having been done. Most (> 80%) drug intervention development studies fail to reach the 'Phase III' trial stage.[36] Public health interventions have tended to go straight to an RCT without piloting which may contribute to challenges in demonstrating effectiveness.[11] There are of course other factors that influence trial findings, including trial methods and conduct, however our question was specifically determined to explore what, if any, relationship existed between intervention development and outcomes and not in the effectiveness of particular interventions.

A strength of our study is the use of integrative mixed methods analysis which has enabled us to explore the relationship between development work and outcome. This rarely used approach in evidence synthesis[37] has given us a unique insight that would not have been possible using a quantitative or qualitative analysis alone. A limitation of our work could be the focus on a single UK funding stream which does not necessarily reflect the body of research funded from other sources and therefore the quality of intervention development work is not necessarily generalizable. However, the NIHR HTA programme is the single largest funder of randomised controlled trials of applied health research in the UK. They publish detailed monographs of their funded studies, along with protocols and other supporting publications that provide a detailed and rich source of data beyond what would normally be available in journal-based peer reviewed publications alone. We were able to retain the essence and nuances of the qualitative data whilst developing categorical ratings of quality to help us better explore the relationship between development work and treatment success.

Our findings are similar to those of Dent and Raftery[9] in relation to those trials showing a benefit who reported 19% (16/85) of studies found in favour of the new intervention. It has been suggested that a 50% success rate is a good investment for healthcare research,[38] however, our findings indicate that the studies we reviewed fell well below this. In contrast, we observed a considerably larger proportion of true negative studies (8/15; 53%) compared with 19/85 (22%) reported by Dent and Raftery.[9] The

For peer review only - http://bmjopen.bmj.com/site/about/guidelines.xhtml

difference is even greater when compared with a review of cancer trials in the USA where only 2% of trials found a true negative outcome.[10] The reasons for the differences are unclear but could include the pragmatic nature of HTA funded trials and the relative smaller effect sizes often associated with trials of rehabilitation.[39]

It has been recently suggested that RCTs should only be undertaken if they are justified both scientifically and ethically by having a clear hypothesis and established uncertainty[40] and our findings support that by way of good quality intervention development work. Our findings also align with the elements suggested to be key for developing interventions and reducing research waste by increasing the likelihood of success[41] which will form a comprehensive supplement to the development phase of the updated MRC guidance on developing and evaluating interventions due for publication in 2019. The NIHR HTA is publically funded and by increasing effort and focus on developing rehabilitation and other interventions in the future researchers and funding bodies could increase the possibility of a definitive trial reporting beneficial findings after much investment of time and public money.

CONCLUSIONS

Despite much research effort and funding, only four out of fifteen evaluations of 'new' rehabilitation interventions funded by the NIHR HTA programme were found to be unequivocally effective. Most studies reported no difference in outcome between study arms. We have used mixed methods research to explore the relationship between intervention development work and treatment success and developed a method of assessing the quality of this work which suggests comprehensive intervention development work may have a positive relationship with treatment success.

RECOMMENDATIONS

As this was an exploratory study, further work should be undertaken to establish the validity of quality assessment of intervention development work. This said, researchers and funding agencies should not undervalue the potential benefit of high quality intervention development work prior to definitive randomised controlled trials to reduce the likelihood of a null outcome and improve current rates of treatment success.

ACKNOWLEDGEMENT

This work was supported by the NIHR Collaboration for Leadership in Applied Health Research and Care South West Peninsula (PenCLAHRC). The views expressed are those of the authors and not necessarily the NHS, the NIHR or the Department of Health and Social Care.

COMPETING INTERESTS

None

DATA SHARING STATEMENT

No additional data are available.

AUTHOR CONTRIBUTION

For peer review only - http://bmjopen.bmj.com/site/about/guidelines.xhtml

For peer review only

VG: Conception and design, data collection, analysis and interpretation, drafting and approving the manuscript;

JH: Design, data collection, analysis and interpretation, drafting and approving the manuscript;

JF: Data collection, analysis, revising and approving the manuscript;

KF: Data collection, revising and approving the manuscript;

CP: Data collection, revising and approving the manuscript;

DR: Conception, revising and approving the manuscript.

REFERENCES

1. World Health Organization. Rehabilitation in health systems. Geneva, 2017.
2. De Souza L. Theories about therapies are underdeveloped. *Physiotherapy Research International* 1998;3(3):iv-vi.
3. Hislop HJ. Tenth Mary McMillan lecture. The not-so-impossible dream. *Phys Ther* 1975;55(10):1069-80. [published Online First: 1975/10/01]
4. Mayo N, Kaur N, Barbic S, et al. How have research questions and methods used in clinical trials published in Clinical Rehabilitation changed over the last 30 years? *Clinical Rehabilitation* 2016;30(9):847-64.
5. Medical Research Council. A framework for development and evaluation of RCT's for complex interventions to improve health. London: Medical Research Council, 2000.
6. Medical Research Council. Developing and evaluating complex interventions: new guidance. London: Medical Research Council, 2008.
7. Richards D. The complex interventions framework. In: Richards D, Hallberg I, eds. Complex interventions in health: an overview of research methods. Abingdon: Routledge 2015:5.
8. Chalmers I, Glasziou P. Avoidable waste in the production and reporting of research evidence. *Lancet* 2009;374(9683):86-9. doi: 10.1016/S0140-6736(09)60329-9 [published Online First: 2009/06/16]
9. Dent L, Raftery J. Treatment success in pragmatic randomised controlled trials: a review of trials funded by the UK Health Technology Assessment programme. *Trials* 2011;12:109. doi: 10.1186/1745-6215-12-109 [published Online First: 2011/05/06]
10. Djulbegovic B, Kumar A, Soares HP, et al. Treatment success in cancer: new cancer treatment successes identified in phase 3 randomized controlled trials conducted by the National Cancer Institute-sponsored cooperative oncology groups, 1955 to 2006. *Arch Intern Med* 2008;168(6):632-42. doi: 10.1001/archinte.168.6.632 [published Online First: 2008/03/26]
11. Hallingberg B, Turley R, Segrott J, et al. Exploratory studies to decide whether and how to proceed with full-scale evaluations of public health interventions: a systematic review of guidance. *Pilot Feasibility Stud* 2018;4:104. doi: 10.1186/s40814-018-0290-8 [published Online First: 2018/06/02]
12. Moore L, Hallingberg B, Wight D, et al. Exploratory studies to inform full-scale evaluations of complex public health interventions: the need for guidance. *J Epidemiol Community Health* 2018;72(10):865-66. doi: 10.1136/jech-2017-210414 [published Online First: 2018/07/22]
13. Barbour RS. The case for combining qualitative and quantitative approaches in health services research. *J Health Serv Res Policy* 1999;4(1):39-43. doi: 10.1177/135581969900400110 [published Online First: 1999/05/27]
14. Arain M, Campbell MJ, Cooper CL, et al. What is a pilot or feasibility study? A review of current practice and editorial policy. *BMC Medical Research Methodology* 2010;10(1):67. doi: 10.1186/1471-2288-10-67

For peer review only - http://bmjopen.bmj.com/site/about/guidelines.xhtml

15. Mohler R, Kopke S, Meyer G. Criteria for Reporting the Development and Evaluation of Complex Interventions in healthcare: revised guideline (CReDECI 2). *Trials* 2015;16:204. doi: 10.1186/s13063-015-0709-y [published Online First: 2015/05/04]
16. Hoffmann TC, Glasziou PP, Boutron I, et al. Better reporting of interventions: template for intervention description and replication (TIDieR) checklist and guide. *BMJ* 2014;348:g1687. doi: 10.1136/bmj.g1687 [published Online First: 2014/03/13]
17. Bowen A, Hesketh A, Patchick E, et al. Clinical effectiveness, cost-effectiveness and service users' perceptions of early, well-resourced communication therapy following a stroke: a randomised controlled trial (the ACT NoW Study). *Health Technol Assess* 2012;16(26):1-160. doi: 10.3310/hta16260 [published Online First: 2012/05/23]
18. Clarke CE, Patel S, Ives N, et al. Clinical effectiveness and cost-effectiveness of physiotherapy and occupational therapy versus no therapy in mild to moderate Parkinson's disease: a large pragmatic randomised controlled trial (PD REHAB). *Health Technol Assess* 2016;20(63):1-96. doi: 10.3310/hta20630 [published Online First: 2016/09/02]
19. Epps H, Ginnelly L, Utley M, et al. Is hydrotherapy cost-effective? A randomised controlled trial of combined hydrotherapy programmes compared with physiotherapy land techniques in children with juvenile idiopathic arthritis. *Health Technol Assess* 2005;9(39):iii-iv, ix-x, 1-59. [published Online First: 2005/09/27]
20. Glazener C, Boachie C, Buckley B, et al. Conservative treatment for urinary incontinence in Men After Prostate Surgery (MAPS): two parallel randomised controlled trials. *Health Technol Assess* 2011;15(24):1-290, iii-iv. doi: 10.3310/hta15240 [published Online First: 2011/06/07]
21. Avert Trial Collaboration group. Efficacy and safety of very early mobilisation within 24 h of stroke onset (AVERT): a randomised controlled trial. *Lancet* 2015;386(9988):46-55. doi: 10.1016/S0140-6736(15)60690-0 [published Online First: 2015/04/22]
22. Jolly K, Taylor R, Lip GY, et al. The Birmingham Rehabilitation Uptake Maximisation Study (BRUM). Home-based compared with hospital-based cardiac rehabilitation in a multi-ethnic population: cost-effectiveness and patient adherence. *Health Technol Assess* 2007;11(35):1-118. [published Online First: 2007/09/05]
23. Lamb SE, Williams MA, Williamson EM, et al. Managing Injuries of the Neck Trial (MINT): a randomised controlled trial of treatments for whiplash injuries. *Health Technol Assess* 2012;16(49):iii-iv, 1-141. doi: 10.3310/hta16490 [published Online First: 2012/12/18]
24. Logan PA, Armstrong S, Avery TJ, et al. Rehabilitation aimed at improving outdoor mobility for people after stroke: a multicentre randomised controlled study (the Getting out of the House Study). *Health Technol Assess* 2014;18(29):vii-viii, 1-113. doi: 10.3310/hta18290 [published Online First: 2014/05/09]
25. McCarthy CJ, Mills PM, Pullen R, et al. Supplementation of a home-based exercise programme with a class-based programme for people with osteoarthritis of the knees: a randomised controlled trial and health economic analysis. *Health Technol Assess* 2004;8(46):iii-iv, 1-61. [published Online First: 2004/11/06]
26. Sackley CM, Walker MF, Burton CR, et al. An Occupational Therapy intervention for residents with stroke-related disabilities in UK Care Homes (OTCH): cluster randomised controlled trial with economic evaluation. *Health Technol Assess* 2016;20(15):1-138. doi: 10.3310/hta20150 [published Online First: 2016/03/02]
27. Underwood M, Lamb SE, Eldridge S, et al. Exercise for depression in care home residents: a randomised controlled trial with cost-effectiveness analysis (OPERA). *Health Technol Assess* 2013;17(18):1-281. doi: 10.3310/hta17180 [published Online First: 2013/05/02]
28. Vickers AJ, Rees RW, Zollman CE, et al. Acupuncture of chronic headache disorders in primary care: randomised controlled trial and economic analysis. *Health Technol Assess* 2004;8(48):iii, 1-35. [published Online First: 2004/11/06]
29. Waterhouse JC, Walters SJ, Oluboyede Y, et al. A randomised 2 x 2 trial of community versus hospital pulmonary rehabilitation, followed by telephone or conventional follow-up. *Health*

For peer review only - http://bmjopen.bmj.com/site/about/guidelines.xhtml

Technol Assess 2010;14(6):i-v, vii-xi, 1-140. doi: 10.3310/hta14060 [published Online First: 2010/02/12]

30. Weindling AM, Cunningham CC, Glenn SM, et al. Additional therapy for young children with spastic cerebral palsy: a randomised controlled trial. *Health Technol Assess* 2007;11(16):iii-iv, ix-x, 1-71. [published Online First: 2007/04/28]
31. Williams MA, Williamson EM, Heine PJ, et al. Strengthening And stretching for Rheumatoid Arthritis of the Hand (SARAH). A randomised controlled trial and economic evaluation. *Health Technol Assess* 2015;19(19):1-222. doi: 10.3310/hta19190 [published Online First: 2015/03/10]
32. Underwood M, Eldridge S, Lamb S, et al. The OPERA trial: protocol for a randomised trial of an exercise intervention for older people in residential and nursing accommodation. *Trials* 2011;12:27. doi: 10.1186/1745-6215-12-27
33. Moher D, Schulz KF, Altman DG. The CONSORT statement: revised recommendations for improving the quality of reports of parallel group randomised trials. *Lancet* 2001;357:1191-94.
34. Boote J, Baird W, Sutton A. Public involvement in the systematic review process in health and social care: a narrative review of case examples. *Health Policy* 2011;102(2-3):105-16. doi: 10.1016/j.healthpol.2011.05.002 [published Online First: 2011/06/07]
35. O'Cathain A, Croot L, Sworn K, et al. Taxonomy of approaches to developing interventions to improve health: a systematic methods overview. *Pilot Feasibility Stud* 2019;5:41. doi: 10.1186/s40814-019-0425-6 [published Online First: 2019/03/30]
36. Arrowsmith J. Trial watch: Phase II failures: 2008-2010. *Nat Rev Drug Discov* 2011;10(5):328-9. doi: 10.1038/nrd3439 [published Online First: 2011/05/03]
37. Petticrew M, Rehfuess E, Noyes J, et al. Synthesizing evidence on complex interventions: how meta-analytical, qualitative, and mixed-method approaches can contribute. *J Clin Epidemiol* 2013;66(11):1230-43. doi: 10.1016/j.jclinepi.2013.06.005 [published Online First: 2013/08/21]
38. Djulbegovic B. Acknowledgment of uncertainty: a fundamental means to ensure scientific and ethical validity in clinical research. *Curr Oncol Rep* 2001;3(5):389-95. [published Online First: 2001/08/08]
39. Angst F, Aeschlimann A, Stucki G. Smallest detectable and minimal clinically important differences of rehabilitation intervention with their implications for required sample sizes using WOMAC and SF-36 quality of life measurement instruments in patients with osteoarthritis of the lower extremities. *Arthritis and rheumatism* 2001;45(4):384-91. doi: 10.1002/1529-0131(200108)45:4<384::AID-ART352>3.0.CO;2-0 [published Online First: 2001/08/15]
40. De Meulemeester J, Fedyk M, Jurkovic L, et al. Many randomized clinical trials may not be justified: a cross-sectional analysis of the ethics and science of randomized clinical trials. *J Clin Epidemiol* 2018;97:20-25. doi: 10.1016/j.jclinepi.2017.12.027 [published Online First: 2018/01/07]
41. Bleijenberg N, de Man-van Ginkel JM, Trappenburg JCA, et al. Increasing value and reducing waste by optimizing the development of complex interventions: Enriching the development phase of the Medical Research Council (MRC) Framework. *International journal of nursing studies* 2018;79:86-93. doi: 10.1016/j.ijnurstu.2017.12.001 [published Online First: 2017/12/09]

Figure Legends:

Figure 1 Study selection

For peer review only

For peer review only - http://bmjopen.bmj.com/site/about/guidelines.xhtml

Figure 2 Classification of Primary Outcome

Figure 3 Treatment success of included trials based on 95% Confidence Intervals and Minimum Clinically Important Difference from sample size calculation (d)

For peer review only

For peer review only - http://bmjopen.bmj.com/site/about/guidelines.xhtml

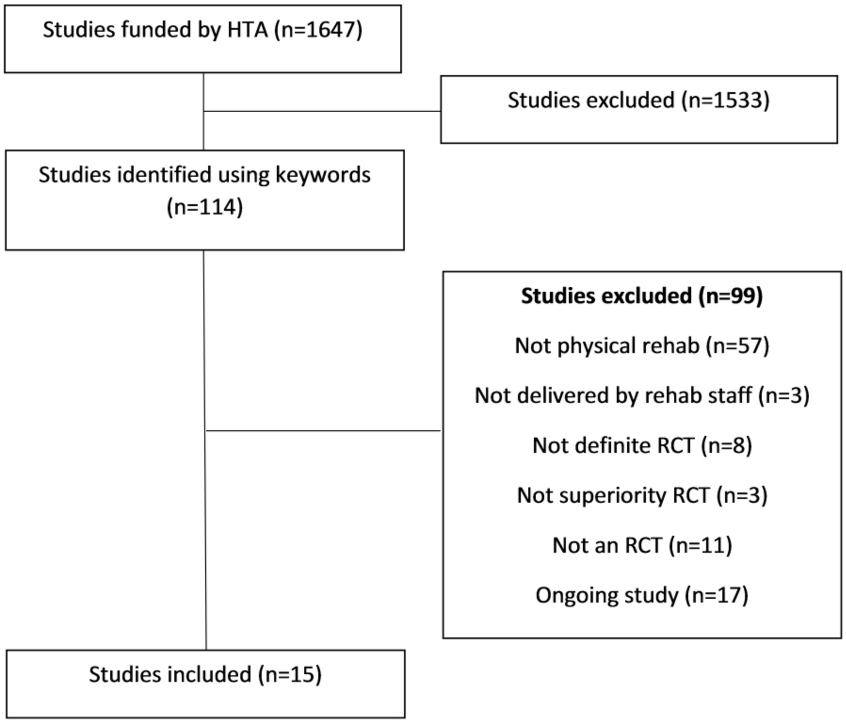

Figure 1 Study selection

Figure 1 Study selection

89x82mm (300 x 300 DPI)

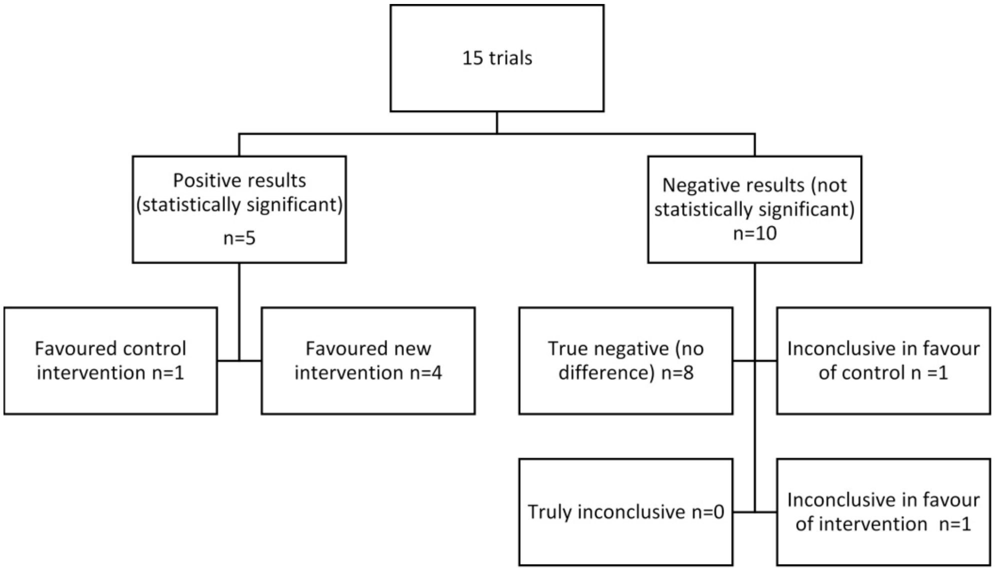

Figure 2 Classification of primary outcome

Figure 2 Classification of Primary Outcome

89x66mm (300 x 300 DPI)

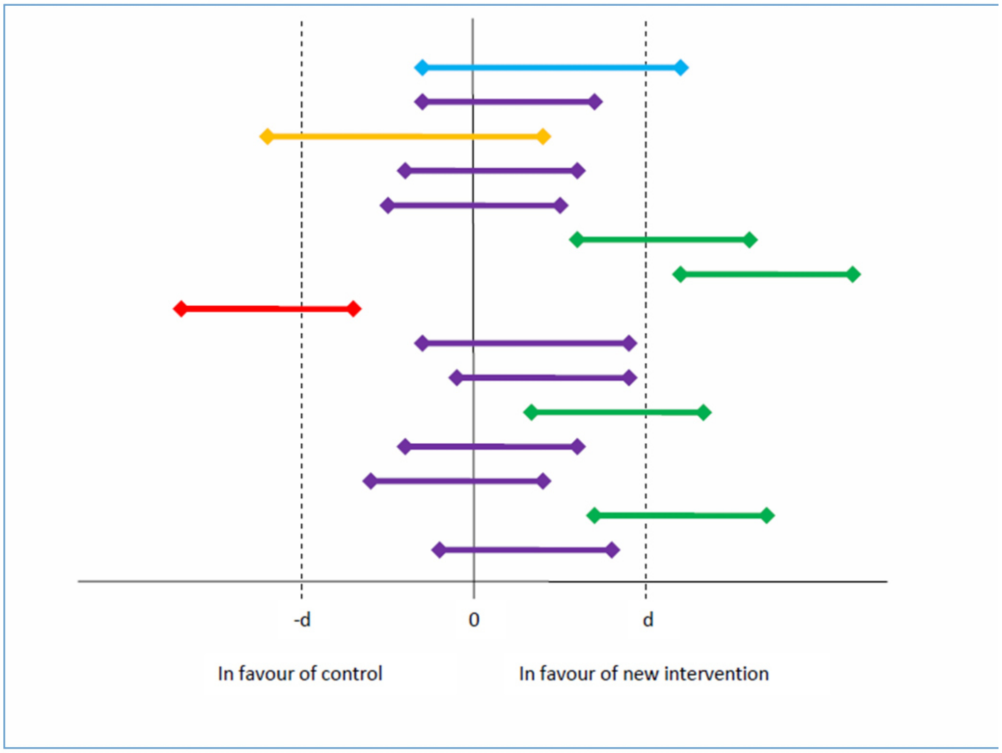

Figure 3 Treatment success of included trials based on 95% Confidence Interval and Minimal Clinically Important Difference from trial sample size calculation (d)

Key: *Green = statistically significantly in favour of intervention; Red = statistically significantly in favour of control; Blue = Inconclusive in favour of intervention; Yellow = Inconclusive in favour of control; Purple = True negative (no difference)*

Figure 3 Treatment success of included trials based on 95% Confidence Intervals and Minimum Clinically Important Difference from sample size calculation (d)

87x89mm (300 x 300 DPI)

Intervention development and treatment success in UK Health Technology Assessment funded trials of physical rehabilitation: a mixed methods analysis

Journal:	*BMJ Open*
Manuscript ID	bmjopen-2018-026289.R2
Article Type:	Research
Date Submitted by the Author:	08-Jul-2019
Complete List of Authors:	Goodwin, Victoria; University of Exeter Medical School<br>Hill, Jacqueline; University of Exeter Medical School<br>Fullam, James; University of Exeter Medical School<br>Finning, Katie; University of Exeter Medical School<br>Pentecost, C; University of Exeter Medical School<br>Richards, David; University of Exeter, Medical School
**Primary Subject Heading**:	Health services research
Secondary Subject Heading:	Rehabilitation medicine
Keywords:	rehabilitation, intervention development, mixed methods, randomised controlled trials

SCHOLARONE™
Manuscripts

Intervention development and treatment success in UK Health Technology Assessment funded trials of physical rehabilitation: a mixed methods analysis

*Victoria A Goodwin, University of Exeter, Exeter UK

Jacqueline J Hill, University of Exeter, Exeter UK

James Fullam, University of Exeter, Exeter UK

Katie Finning, University of Exeter, Exeter UK

Claire Pentecost, University of Exeter, Exeter UK

David A Richards, University of Exeter, Exeter UK

* Corresponding author: 2.26 South Cloisters, University of Exeter Medical School, St Luke's Campus, Magdalen Road, Exeter EX1 2LU. Telephone 01392 722745 v.goodwin@exeter.ac.uk

Keywords

Rehabilitation, randomised controlled trials, quality, intervention development, mixed methods

For peer review only

Abstract

Objectives: Physical rehabilitation is a complex process and trials of rehabilitation interventions are increasing in number but often report null results. This study aimed to establish treatment success rates in physical rehabilitation trials funded by the National Institute of Health Research Health Technology Assessment (NIHR HTA) programme and examine any relationship between treatment success and the quality of intervention development work undertaken.

Design: Mixed methods study

Setting: UK

Methods:

The NIHR HTA portfolio was searched for all completed definitive randomised controlled trials of physical rehabilitation interventions from inception to July 2016. Treatment success was categorised according to criteria developed by Djulbegovic and colleagues. Detailed textual data regarding any intervention development work were extracted from trial reports and supporting publications and informed the development of quality ratings. Mixed methods integrative analysis was undertaken to explore the relationship between quantitative and qualitative data using joint displays.

Results: Fifteen trials were included in the review. Five reported a definitive finding, four of which were in favour of the 'new' intervention. Eight trials reported a true negative (no difference) outcome. Integrative analysis indicated those with lower quality intervention development work were less likely to report treatment success.

Conclusions: Despite much effort and funding, most physical rehabilitation trials report equivocal findings. Greater focus on high quality intervention development may reduce the likelihood of a null result in the definitive trial, alongside high quality trial methods and conduct.

Strengths and limitations of this study:

- To our knowledge, this study is the first to use mixed methods integrative analyses to explore the relationship between quality of intervention development work and treatment success.
- Using the NIHR HTA Journal monographs, published protocols and other supporting publications for each study together provided a detailed and rich source of data beyond what would be found in a single traditional journal publication.
- The study reviewed randomised controlled trials of physical rehabilitation from a single UK funder as an exemplar and therefore findings may not be representative of other complex interventions or other funding bodies.

For peer review only

BACKGROUND

Rehabilitation is *"a set of interventions designed to optimise function and reduce disability in individuals with health conditions in interaction with their environment".*[1] and is an essential aspect of healthcare provision. By its very nature rehabilitation in clinical practice is an individually focused, complex activity, involving interventions that are multi-faceted and often implicit in nature[2] and as such, historically, this has been viewed as a barrier to undertaking research.[3] This said, there is a growing body of randomised controlled trials (RCTs) of rehabilitation, suggesting that these challenges can be overcome.[4] This may, in part, be supported by the publication of the Medical Research Council (MRC) Framework for developing and evaluating complex interventions.[5 6]

The MRC framework was developed to optimise the likelihood that new interventions are not rejected as being ineffective when inadequate effort has been made in the development of the intervention.[7] Likewise, Chalmers and Glasziou[8] highlighted the importance of avoiding research waste and recommended that sufficient effort is made to ensure the relevant research questions are identified and addressed using high quality research methods. However, there appears to have been no formal evaluation of the impact of using the development component of the framework on trial outcomes and whether we are observing evidence of effective interventions being developed.

Previous UK[9] and USA[10] reviews synthesised successful and non-successful treatment outcomes from trials of new interventions in order to assess the equipoise principle and to understand what return has been achieved on the investment made by those taking part in the trials, researchers and funders. Dent and Raftery[9] reported 24% (20/85) primary outcome comparisons as having a positive result, of which 16/85 (19%) were in favour of the new intervention, with 19/85 (22%) comparisons reporting a true negative outcome. However, these authors did not focus on rehabilitation interventions, nor did they seek to understand factors that may impact on treatment success, such as the quality or intensity of intervention development pre-trial procedures. Informal discussions with colleagues in the UK and internationally noted that an increasing number of publically funded, large RCTs evaluating physical rehabilitation interventions had reported null findings. Similar concerns have been reported in studies of public health interventions.[11 12] Our study, therefore, sought to assess this observation and also explore whether intervention development activities contributed to treatment success using the National Institute of Health Research Health Technology Assessment programme (NIHR HTA) as an exemplar.

We aimed to use data from the NIHR HTA to:

(a) Establish the treatment outcomes of funded RCTs of physical rehabilitation;

(b) Establish how many new interventions were found to be effective;

(c) Examine what work had been done in terms of developing the new intervention;

(d) Examine the relationship between (a) and (c).

We adopted a mixed methods approach to address the study aims. Although evidence of using integrative mixed methods approaches in synthesising evidence on complex interventions is limited, mixing together qualitative and quantitative data can generate understanding that has the potential to be greater than the sum of the individual parts.[13]

For peer review only - http://bmjopen.bmj.com/site/about/guidelines.xhtml

METHODS

Design

We undertook a review of NIHR HTA funded randomised controlled trials of physical rehabilitation interventions using narrative synthesis of outcomes and mixed methods analysis of the relationship between intervention development and categorical treatment outcomes using joint displays.

Patient and Public Involvement

Patients and the public were not involved in this study.

Data sources and inclusion criteria

We included superiority randomised controlled trials of physical rehabilitation funded by the NIHR HTA programme. The interventions could be delivered by a single profession or be multi-professional. The NIHR HTA programme is the leading public funding source for randomised controlled trials (RCTs) in the UK and trials of rehabilitation are increasingly part of the portfolio. We only included completed RCTs whose main trial findings were reported in an HTA monograph or peer-reviewed publication in order to establish treatment success. We excluded: pilot and feasibility RCTs as they do not aim to assess the efficacy or effectiveness of interventions;[14] studies where the interventions were primarily psychological or cognitive as the focus of the study was physical rehabilitation; where the primary outcome findings were not reported with a 95% confidence interval (CI) as these data were required to assess treatment success.

Search and screening

We searched the HTA Project Portfolio (since superseded by the NIHR Journals Library https://www.journalslibrary.nihr.ac.uk/#/) from inception to July 2016 using the following keywords: physiotherap*OR occupational therap* OR speech and language therap* OR rehabilitation. We removed duplicates and then titles and scientific abstracts were reviewed for potential inclusion by one person and checked by a second. Subsequently full text reports were screened for inclusion by one person and checked by a second. Any disagreements were discussed and agreed with a third person.

Data extraction

All data were extracted by one person and checked by a second. Discrepancies were discussed and resolved with a third person.

Quantitative Trial data: Data extracted from each trial publication included trial design, target population, health categories (using the Health Research Classification System), primary outcome(s) and time point, minimal important clinical difference (MCID) or percentage change that the trial aimed to detect, planned and achieved sample size, and primary outcome results with 95% CI. We also recorded the professional background of the Chief Investigator and amount of funding awarded.

Qualitative Intervention development data: Using the revised version of Criteria for Reporting the Development and Evaluation of Complex Interventions (CReDECI 2)[15] and the Template for Intervention Description and Replication checklist (TIDieR)[16] as frameworks we extracted all available

For peer review only - http://bmjopen.bmj.com/site/about/guidelines.xhtml

documentary (qualitative) data from the body of the text regarding intervention development, including descriptions of underlying theory, intervention components and reasons for selection, intended interactions between components, contextual considerations, piloting of intervention and impact of definitive intervention to be evaluated, control components, planned intervention delivery and materials. Where additional supporting publications were cited, such as a protocol or intervention development studies, we used these as additional sources of documentary data.

Data analysis

We used summary statistics to describe the characteristics of the included studies. We categorised primary outcome findings into one of six treatment outcome categories as described by Djulbegovic and colleagues,[10] these being: 1) statistically significant in favour of the new treatment, 2) statistically significant in favour of the control treatment 3) true negative, 4) truly inconclusive, 5) inconclusive in favour of new treatment or 6) inconclusive in favour of the control treatment. This was achieved by comparing the 95% confidence interval for the difference in primary outcome to the difference specified in the sample size calculation.[9] If the 95% confidence interval excluded a meaningful difference in either direction, implying the treatments have similar effects, the results were categorised as true negative. If the 95% confidence interval included a meaningful difference in either direction (i.e. trial failed to answer the primary question), the results were categorised as being truly inconclusive.

Where a single primary outcome and primary time point were not explicitly identified we utilised the following hierarchy to determine which primary outcome would be used in the analysis:

- Explicitly defined primary outcome
- Outcome used in power calculation
- Main outcome stated in trial objectives
- First outcome reported in sample size calculation

If a primary time point was not reported we used the first follow up time point as this is when we would expect the intervention to have had the greatest effect.

Our preliminary analysis of the qualitative documentary data involved the reading and re-reading of source documents and the extracted descriptions to consolidate our understanding of the development work undertaken in each study. Using a reflective and iterative process we undertook thematic analysis to distil, structure and make sense of intervention development activity by coding and organising data into themes and subthemes. Each theme and sub-theme provided a coherent description of the development work undertaken for each study, which were then synthesised into short descriptors to allow us to produce summary tables. The summary tables comprised a row for each study with columns for each theme and, where relevant, each subtheme. A second researcher checked, discussed and refined descriptors to ensure accuracy. From these descriptions we then developed descriptive ratings on the quality of the intervention development. Depending on the nature of the data, ratings were categorised and the iterative process involved two researchers refining and checking ratings to ensure they reflected the summary data from each study. In order to provide a visual representation of the quality of intervention development work these ratings were then converted to a quality coding to indicate high quality, some or unclear quality or limited quality. For example under co-design the highest quality rating was given when the intervention was co-

For peer review only - http://bmjopen.bmj.com/site/about/guidelines.xhtml

designed with *both* clinical and service user input, a middle rating when *either* clinicians or service users were involved, and the lowest quality rating when *neither* clinicians nor service users were involved.

To examine the relationship between intervention development and treatment success, we applied mixed methods analytical techniques in novel ways. For each study, we combined ratings derived from the qualitative data on intervention development with the quantitative data on treatment outcomes in a joint display.

RESULTS

We included fifteen RCTs (Figure 1),[17-31] of which thirteen used a two-arm, parallel RCT design, one was a two-arm cluster RCT and one was a four-arm factorial design (of which only two arms related to physical rehabilitation). Table 1 provides a summary of the population, intervention, control and outcomes for each study. The combined target sample size was 7548 participants, 7834 of whom provided primary outcome data, although three studies[19 29 30] were considerably below their target sample size at the primary time point. Five primary outcomes were symptom-based or clinical outcomes, seven were functional measures, two were combined measures and one assessed quality of life. Primary time points varied from immediately post-intervention to one year (median 6 months). The health categories were: Stroke (n=4), Neurological conditions (n=2), Inflammatory/Immune system disorders (n=2), Respiratory (n=1), Musculoskeletal (n=1), Cardiovascular (n=1), Mental Health (n=1), Accident/injuries (n=1), Renal/urogenital (n=1) and other (n=1). Seven interventions were delivered by physiotherapists, one by occupational therapists, one by speech and language therapists, one by nurses, two could be delivered by either a physiotherapist or a nurse, two could be delivered by a physiotherapist or an occupational therapist and one was delivered by both a physiotherapist and an occupational therapist. The Chief Investigators leading the studies were physicians (n=7), physiotherapists (n=5), occupational therapists (n=1), psychologists (n=1) and methodologists (n=1). The total amount of research funding awarded was £12,515,823.

One third of studies (5/15) reported a definitive finding in favour of one of the treatment arms - four studies in favour of the new treatment, one in favour of the control. Of those with negative results, eight studies reported a true negative (no difference) outcome, one was inconclusive in favour of the new treatment, and one inconclusive in favour of the control treatment (Figures 2 and 3).

Qualitative data informed two themes and ten sub-themes which enabled us to develop data-driven quality ratings:

1. *Preparatory work* (Need for the study, underpinning theory for the intervention, co-design, context considerations and intervention piloting)
2. *Intervention and control* (Intervention content and dose, individual tailoring, adherence strategies, standardised training, control content and dose)

Table 2 provides examples of summary data underpinning each rating with Table 3 describing the quality rating for each study in chronological order. Table 4 presents the integrative qualitative and quantitative analysis using a joint display. No single study was deemed to be high quality in each sub-theme. This said, the two best rated studies reported only expert clinical input into co-designing the

For peer review only - http://bmjopen.bmj.com/site/about/guidelines.xhtml

intervention with a lack of clear patient and public involvement, however, they reported a definitive trial outcome in favour of the new intervention. There does not appear to be a single aspect of intervention development driving study outcomes. This said, those with lower quality development work appear more likely to show no difference in outcomes compared with those with higher quality development work. Some areas of intervention development appear to be improving with time, these being articulating a clear need and theoretical underpinning, co-design, piloting and descriptions of intervention and control components.

For peer review only

For peer review only - http://bmjopen.bmj.com/site/about/guidelines.xhtml

Table 1 Summary of included studies

Author (year published)	Funding awarded (£)	Population (target sample size/number of participants with primary outcome data)	Intervention	Control	Primary Outcome (MCID or % change study aimed to detect)
McCarthy et al (2004)	218,517	People with knee osteoarthritis (n=152/200)	Twice weekly exercise group for 8 weeks plus home exercises	Home exercises	Aggregate Locomotor Function score (4 seconds)
Vickers et al (2004)	161,532	People with chronic headache (n=288/301)	Up to 12 acupuncture treatments plus usual care	Usual care from General Practitioner	Weekly headache score (35% reduction)
Epps et al (2005)	152,011	Children with juvenile arthritis (n=200/74)	8 hydrotherapy and 8 land based sessions over 2 weeks followed by weekly/fortnightly hydrotherapy for 2 months	16 land based exercise sessions over 2 weeks followed by weekly or fortnightly land based exercise sessions	Disease status calculated from Childhood Health Assessment Questionnaire (CHAQ), physicians' global assessment of disease activity, parents' global assessment of overall well-being, number of joints with limited ROM, number of active joints, erythrocyte sedimentation rate (30% improvement on 3 measures with < 30% deterioration on remaining 3 measures)
Weindling et al (2007)	334,093	Children with cerebral palsy (n=153/76)	Regular physiotherapy (usual care) plus additional weekly session from physiotherapy assistant for 6 months	Usual care (regular physiotherapy)	Gross Motor Function Measure (14 points)
Jolly et al (2007)	480,612	People with myocardial infarction or revascularisation (n=450/487)	Home-based self-help manual plus up to 3 face to face and 1 phone call support over 12 weeks	Centre-based cardiac rehabilitation	Incremental shuttle walk test (6 shuttles); Hospital anxiety and depression scale (1.5 points); smoking cessation (20%); blood pressure (6mmHg systolic); serum cholesterol (0.4 mmol/l)
Waterhouse et al (2010)	460,543	People with Chronic Obstructive Pulmonary Disease (n=372/162)	Twice weekly community-based pulmonary rehabilitation	Twice weekly hospital-based pulmonary rehabilitation	Endurance Shuttle Walk Test (60% increase in distance walked)
Glazener et al (2011)	1,051,699	Men with incontinence post-prostate surgery (696/788)	Assessment and treatment and exercise over 4 face to face sessions plus advice leaflet	Advice leaflet	Self-reported urinary incontinence (15% reduction in % of people with urinary incontinence)
Bowen et al (2012)	1,457,533	Adults with aphasia or dysarthria after stroke (n=170/153)	Speech and language therapy visits up to 3 sessions per week for up to 16 weeks	Volunteer visits up to 3 sessions per week for up to 16 weeks	Therapy Outcome measure (0.5)
Lamb et al (2012)	755,310	People with whiplash with persistent symptoms (n=422/507)	6 sessions of assessment and treatment/exercise over 8 weeks	Single session of advice	Neck Disability Index (3 points)
Underwood et al (2013)	1,957,884	Care home residents (n=409/493)	Twice weekly exercise group for a year	Depression awareness training for care home staff	Geriatric Depression Scale (17.3% reduction in % of people with depression)
Logan et al (2014)	993,080	People with stroke (n=440/503)	Up to 12 therapy visits to increase outdoor mobility plus verbal/written advice	Verbal/written advice	SF-36 Social function domain (12.5 points)
Williams et al (2015)	976,955	People with rheumatoid arthritis (n=352/438)	6 sessions of exercise plus home exercises over 12 weeks	Single assessment advice session with 2 further optional sessions over 12 weeks (no exercises)	Michigan Hand Outcome Questionnaire (0.3)
AVERT Group (2015)	282,372	People with stroke (n=2104/2083)	3 additional out of bed sessions per day for up to 2 weeks	Usual care	Modified Rankin Scale-(mRS) (7.1% Absolute risk reduction of a mRS score of 3-6)

For peer review only - http://bmjopen.bmj.com/site/about/guidelines.xhtml

Sackley et al (2016)	1,797,676	Care home residents with stroke (n=660/870)	Individualised occupational therapy	No occupational therapy	Barthel Index (2 points)
Clarke et al (2016)	1,436,006	People with Parkinson's (n=680/699)	Up to 8 individualised sessions of Physiotherapy and up to 8 individualised sessions of occupational therapy	No therapy	Nottingham Extended Activities of Daily Living (2.5 points)

For peer review only

For peer review only - http://bmjopen.bmj.com/site/about/guidelines.xhtml

Table 2 Description of themes, subthemes and quality ratings with examples

Theme	Sub-theme	Description of rating	Examples of data supporting rating	Rating
**Preparatory work**	**Need for the study**	Multiple sources of evidence of need for the study e.g. recent systematic review, guidelines, high level reports, commissioned research, national audit	International task force highlighted lack of evidence and need for evaluation. Cochrane review drew similar conclusions.	▲
		Single source of evidence / non-systematic review to support need for study	Old systematic review indicates paucity of high quality research.	■
		Lack of clarity or underpinning evidence regarding need for study	Poor justification for the study. Evidence cited doesn't support the need for this particular study.	●
	**Theoretical underpinning**	Theoretical underpinning described	Physiological and psychological theories underpinning the intervention described in detail.	▲
		Lacks clear theoretical underpinning	No information provided regarding the theoretical basis for the intervention provided.	■
	**Co-design**	Good PPI and expert clinical input	Patients and clinicians helped develop the intervention.	▲
		Good PPI but weak or no expert clinical input / Good clinical input but unclear or no PPI	Clinicians contributed to the intervention development but no indication of service user involvement.	■
		No co-design	No co-design was undertaken to develop the intervention.	●
	**Contextual considerations**	Context considered	The use of different professionals in delivering the intervention reflected the real world situation of how this would occur in practice.	▲
		Context not adequately considered	There was a lack of understanding of relevant context and factors needed for intervention development and delivery.	●
	**Piloting of intervention**	Pilot conducted, evaluated and findings addressed for main evaluation	The pilot data helped refine the intervention for evaluation in the main trial.	▲
		Pilot conducted but findings not clearly addressed in intervention for main evaluation	The pilot work led to a modification of the control intervention but unclear as to whether this also happened for the novel intervention.	■
		No pilot reported	No piloting of intervention reported	●

For peer review only

**Intervention and control**	**Content and dose**	Intervention components and dose clearly described	The content and the dose of the exercise programme was described in detail.	▲
		Intervention components clearly described but dose was not standardised	The content of the programme was well described but no specific dose was prescribed.	■
		Intervention not replicable from description of components and dose	Intervention was based on usual practice and had no protocol or guidance on minimum dose.	●
	**Tailoring**	Formalised assessment to inform tailoring	An assessment tool was used to determine the individuals level of exercise intensity	▲
		Clinical judgement only used to inform tailoring	Therapists used their clinical judgement to individually tailor programmes.	■
		Not adequately reported	Intervention individually tailored but no information as to how this was undertaken.	●
	**Adherence support strategies**	Explicit strategies to support adherence to the intervention clearly reported	Specific adherence strategies described as part of the intervention.	▲
		No clear information regarding adherence support strategies	No information reported regarding adherence strategies.	●
		Supporting adherence is not relevant to the intervention	The intervention was passive and adherence strategies not relevant.	NA
	**Intervention training**	Standardised training in intervention received +/- additional/ongoing support or training	Staff attended a 1.5 day training session and had an additional support session with ongoing contact from research team.	▲
		No standardised intervention training received but staff delivering described to be experienced in the intervention or training of staff unclear/not reported	Staff have post graduate training in the intervention but no study specific training reported.	■
	**Control description**	Active control/attention control/usual care with some standardised components	Control was an active intervention that differed from intervention only in terms of delivery setting.	▲
		Usual care had no standardised components	Control was usual care and was not standardised between sites.	■

Key: ▲ High quality ■ Some/Unclear quality ● Limited quality

For peer review only

For peer review only - http://bmjopen.bmj.com/site/about/guidelines.xhtml

Table 3 Quality of intervention development work ordered by year of publication

Author	Need	Theory	Co-design	Context	Pilot	Intervention Content	Tailored	Adherence strategies	Training delivery	Control description
McCarthy[25]	■	■	●	▲	●	▲	▲	■	■	▲
Vickers[28]	■	■	●	▲	●	●	●	NA	■	■
Epps[19]	■	▲	■	●	■	▲	■	■	■	▲
Weindling[30]	●	▲	●	●	●	●	●	■	■	■
Jolly[22]	●	▲	●	▲	●	▲	■	▲	▲	■
Waterhouse[29]	▲	■	●	●	●	▲	■	▲	■	▲
Glazener[20]	▲	▲	▲	▲	●	▲	▲	▲	▲	▲
Bowen[17]	▲	▲	■	▲	▲	■	▲	▲	■	▲
Lamb[23]	▲	▲	■	▲	▲	▲	▲	▲	▲	▲
Underwood[32]	▲	▲	■	▲	▲	▲	▲	▲	▲	▲
Logan[24]	▲	▲	▲	▲	■	■	▲	▲	▲	▲
AVERT Group[21]	▲	▲	●	▲	■	▲	▲	NA	▲	■
Williams[31]	▲	▲	■	▲	▲	▲	▲	▲	▲	▲
Sackley[26]	▲	▲	■	▲	■	●	▲	▲	■	■
Clarke[18]	●	■	■	▲	●	●	▲	■	■	■

Key:

▲ High quality ■ Some/Unclear quality ● Limited quality

For peer review only

For peer review only - http://bmjopen.bmj.com/site/about/guidelines.xhtml

Table 4 Joint display of treatment success ordered by quality of intervention development work

Author	Need	Theory	Co-design	Context	Pilot	Intervention Content	Tailored	Adherence strategies	Training delivery	Control description	Treatment success
Lamb[23]	▲	▲	■	▲	▲	▲	▲	▲	▲	▲	Statistically significant in favour of intervention
Williams[31]	▲	▲	■	▲	▲	▲	▲	▲	▲	▲	Statistically significant in favour of intervention
Underwood[32]	▲	▲	■	▲	▲	▲	▲	▲	▲	▲	True negative (No difference)
Glazener[20]	▲	▲	▲	▲	●	▲	▲	▲	▲	▲	True negative (No difference)
Logan[24]	▲	▲	▲	▲	■	■	▲	▲	▲	▲	True negative (No difference)
Bowen[17]	▲	▲	■	▲	▲	■	▲	▲	■	▲	Inconclusive in favour of intervention
AVERT Group[21]	▲	▲	●	▲	■	▲	▲	NA	▲	■	Statistically significant in favour of control
Sackley[26]	▲	▲	■	▲	■	●	▲	▲	■	■	True negative (No difference)
Jolly[22]	●	▲	●	▲	●	▲	■	▲	▲	■	True negative (No difference)
McCarthy[25]	■	■	●	▲	●	▲	▲	■	■	▲	Statistically significant in favour of intervention
Waterhouse[29]	▲	■	●	●	●	▲	■	▲	■	▲	True negative (No difference)
Epps[19]	■	▲	■	●	■	▲	■	■	■	▲	Inconclusive in favour of control
Clarke[18]	●	■	■	▲	●	●	▲	■	■	■	True negative (No difference)
Vickers[28]	■	■	●	▲	●	●	●	NA	■	■	Statistically significant in favour of intervention
Weindling[30]	●	▲	●	●	●	●	●	■	■	■	True negative (No difference)

Key:

▲ High quality ■ Some/Unclear quality ● Limited quality

For peer review only

For peer review only - http://bmjopen.bmj.com/site/about/guidelines.xhtml

DISCUSSION

Physical rehabilitation research targets a broad population although we found that studies for people with stroke to be the most common (n=4). We established that only one third (5/15) of the randomised controlled trials of physical rehabilitation funded by the NIHR HTA programme successfully demonstrated a statistically significant effect for one of the randomised groups in each trial. Four (27%) trials found an effect in favour of the 'new' intervention. Although we would not expect all studies to demonstrate effectiveness in favour of the 'new' intervention, the equipoise principle implies that there would be no difference between the proportion of studies favouring intervention or control[9]. However, this doesn't account for a null outcome. We were able to use contemporary research methods to develop an assessment of the quality of development work and assessed the included trials to be of varied quality in terms of intervention development work. In general, we found that comprehensive intervention development may have a positive relationship with treatment success. Two studies[23 31] with high quality intervention development reported treatment success although two older[25 28] and possibly less well reported trials also reported effective interventions. Developments in complex intervention evaluation[5], reporting standards[16 33] and involving patients and the public in research[34] have occurred since the inception of the HTA programme and as such some development work may have been undertaken but not reported in the older studies. A recent overview of approaches to developing interventions noted the absence of patient and public involvement[35]. In addition, there was limited evidence of piloting the intervention prior to proceeding to the full trial with only four studies reporting this having been done. Most (> 80%) drug intervention development studies fail to reach the 'Phase III' trial stage.[36] Public health interventions have tended to go straight to an RCT without piloting which may contribute to challenges in demonstrating effectiveness.[11] There are of course other factors that influence trial findings, including trial methods and conduct, however our question was specifically determined to explore what, if any, relationship existed between intervention development and outcomes and not in the effectiveness of particular interventions.

A strength of our study is the use of integrative mixed methods analysis which has enabled us to explore the relationship between development work and outcome. This rarely used approach in evidence synthesis[37] has given us a unique insight that would not have been possible using a quantitative or qualitative analysis alone. A limitation of our work could be the focus on a single UK funding stream which does not necessarily reflect the body of research funded from other sources and therefore the quality of intervention development work is not necessarily generalizable. However, the NIHR HTA programme is the single largest funder of randomised controlled trials of applied health research in the UK. They publish detailed monographs of their funded studies, along with protocols and other supporting publications that provide a detailed and rich source of data beyond what would normally be available in journal-based peer reviewed publications alone. We were able to retain the essence and nuances of the qualitative data whilst developing categorical ratings of quality to help us better explore the relationship between development work and treatment success.

Our findings are similar to those of Dent and Raftery[9] in relation to those trials showing a benefit who reported 19% (16/85) of studies found in favour of the new intervention. It has been suggested that a 50% success rate is a good investment for healthcare research,[38] however, our findings indicate that the studies we reviewed fell well below this. In contrast, we observed a considerably larger proportion of true negative studies (8/15; 53%) compared with 19/85 (22%) reported by Dent and Raftery.[9] The

For peer review only - http://bmjopen.bmj.com/site/about/guidelines.xhtml

difference is even greater when compared with a review of cancer trials in the USA where only 2% of trials found a true negative outcome.[10] The reasons for the differences are unclear but could include the pragmatic nature of HTA funded trials and the relative smaller effect sizes often associated with trials of rehabilitation.[39]

It has been recently suggested that RCTs should only be undertaken if they are justified both scientifically and ethically by having a clear hypothesis and established uncertainty[40] and our findings support that by way of good quality intervention development work. Our findings also align with the elements suggested to be key for developing interventions and reducing research waste by increasing the likelihood of success[41] which will form a comprehensive supplement to the development phase of the updated MRC guidance on developing and evaluating interventions due for publication in 2019. The NIHR HTA is publically funded and by increasing effort and focus on developing rehabilitation and other interventions in the future researchers and funding bodies could increase the possibility of a definitive trial reporting beneficial findings after much investment of time and public money.

CONCLUSIONS

Despite much research effort and funding, only four out of fifteen evaluations of 'new' rehabilitation interventions funded by the NIHR HTA programme were found to be unequivocally effective. Most studies reported no difference in outcome between study arms. We have used mixed methods research to explore the relationship between intervention development work and treatment success and developed a method of assessing the quality of this work which suggests comprehensive intervention development work may have a positive relationship with treatment success.

RECOMMENDATIONS

As this was an exploratory study, further work should be undertaken to establish the validity of quality assessment of intervention development work. This said, researchers and funding agencies should not undervalue the potential benefit of high quality intervention development work prior to definitive randomised controlled trials to reduce the likelihood of a null outcome and improve current rates of treatment success.

ACKNOWLEDGEMENT

This work was supported by the NIHR Collaboration for Leadership in Applied Health Research and Care South West Peninsula (PenCLAHRC). The views expressed are those of the authors and not necessarily the NHS, the NIHR or the Department of Health and Social Care.

COMPETING INTERESTS

None

DATA SHARING STATEMENT

No additional data are available.

AUTHOR CONTRIBUTION

For peer review only - http://bmjopen.bmj.com/site/about/guidelines.xhtml

For peer review only

VG: Conception and design, data collection, analysis and interpretation, drafting and approving the manuscript;

JH: Design, data collection, analysis and interpretation, drafting and approving the manuscript;

JF: Data collection, analysis, revising and approving the manuscript;

KF: Data collection, revising and approving the manuscript;

CP: Data collection, revising and approving the manuscript;

DR: Conception, revising and approving the manuscript.

REFERENCES

1. World Health Organization. Rehabilitation in health systems. Geneva, 2017.
2. De Souza L. Theories about therapies are underdeveloped. *Physiotherapy Research International* 1998;3(3):iv-vi.
3. Hislop HJ. Tenth Mary McMillan lecture. The not-so-impossible dream. *Phys Ther* 1975;55(10):1069-80. [published Online First: 1975/10/01]
4. Mayo N, Kaur N, Barbic S, et al. How have research questions and methods used in clinical trials published in Clinical Rehabilitation changed over the last 30 years? *Clinical Rehabilitation* 2016;30(9):847-64.
5. Medical Research Council. A framework for development and evaluation of RCT's for complex interventions to improve health. London: Medical Research Council, 2000.
6. Medical Research Council. Developing and evaluating complex interventions: new guidance. London: Medical Research Council, 2008.
7. Richards D. The complex interventions framework. In: Richards D, Hallberg I, eds. Complex interventions in health: an overview of research methods. Abingdon: Routledge 2015:5.
8. Chalmers I, Glasziou P. Avoidable waste in the production and reporting of research evidence. *Lancet* 2009;374(9683):86-9. doi: 10.1016/S0140-6736(09)60329-9 [published Online First: 2009/06/16]
9. Dent L, Raftery J. Treatment success in pragmatic randomised controlled trials: a review of trials funded by the UK Health Technology Assessment programme. *Trials* 2011;12:109. doi: 10.1186/1745-6215-12-109 [published Online First: 2011/05/06]
10. Djulbegovic B, Kumar A, Soares HP, et al. Treatment success in cancer: new cancer treatment successes identified in phase 3 randomized controlled trials conducted by the National Cancer Institute-sponsored cooperative oncology groups, 1955 to 2006. *Arch Intern Med* 2008;168(6):632-42. doi: 10.1001/archinte.168.6.632 [published Online First: 2008/03/26]
11. Hallingberg B, Turley R, Segrott J, et al. Exploratory studies to decide whether and how to proceed with full-scale evaluations of public health interventions: a systematic review of guidance. *Pilot Feasibility Stud* 2018;4:104. doi: 10.1186/s40814-018-0290-8 [published Online First: 2018/06/02]
12. Moore L, Hallingberg B, Wight D, et al. Exploratory studies to inform full-scale evaluations of complex public health interventions: the need for guidance. *J Epidemiol Community Health* 2018;72(10):865-66. doi: 10.1136/jech-2017-210414 [published Online First: 2018/07/22]
13. Barbour RS. The case for combining qualitative and quantitative approaches in health services research. *J Health Serv Res Policy* 1999;4(1):39-43. doi: 10.1177/135581969900400110 [published Online First: 1999/05/27]
14. Arain M, Campbell MJ, Cooper CL, et al. What is a pilot or feasibility study? A review of current practice and editorial policy. *BMC Medical Research Methodology* 2010;10(1):67. doi: 10.1186/1471-2288-10-67

For peer review only

For peer review only - http://bmjopen.bmj.com/site/about/guidelines.xhtml

15. Mohler R, Kopke S, Meyer G. Criteria for Reporting the Development and Evaluation of Complex Interventions in healthcare: revised guideline (CReDECI 2). *Trials* 2015;16:204. doi: 10.1186/s13063-015-0709-y [published Online First: 2015/05/04]
16. Hoffmann TC, Glasziou PP, Boutron I, et al. Better reporting of interventions: template for intervention description and replication (TIDieR) checklist and guide. *BMJ* 2014;348:g1687. doi: 10.1136/bmj.g1687 [published Online First: 2014/03/13]
17. Bowen A, Hesketh A, Patchick E, et al. Clinical effectiveness, cost-effectiveness and service users' perceptions of early, well-resourced communication therapy following a stroke: a randomised controlled trial (the ACT NoW Study). *Health Technol Assess* 2012;16(26):1-160. doi: 10.3310/hta16260 [published Online First: 2012/05/23]
18. Clarke CE, Patel S, Ives N, et al. Clinical effectiveness and cost-effectiveness of physiotherapy and occupational therapy versus no therapy in mild to moderate Parkinson's disease: a large pragmatic randomised controlled trial (PD REHAB). *Health Technol Assess* 2016;20(63):1-96. doi: 10.3310/hta20630 [published Online First: 2016/09/02]
19. Epps H, Ginnelly L, Utley M, et al. Is hydrotherapy cost-effective? A randomised controlled trial of combined hydrotherapy programmes compared with physiotherapy land techniques in children with juvenile idiopathic arthritis. *Health Technol Assess* 2005;9(39):iii-iv, ix-x, 1-59. [published Online First: 2005/09/27]
20. Glazener C, Boachie C, Buckley B, et al. Conservative treatment for urinary incontinence in Men After Prostate Surgery (MAPS): two parallel randomised controlled trials. *Health Technol Assess* 2011;15(24):1-290, iii-iv. doi: 10.3310/hta15240 [published Online First: 2011/06/07]
21. Avert Trial Collaboration group. Efficacy and safety of very early mobilisation within 24 h of stroke onset (AVERT): a randomised controlled trial. *Lancet* 2015;386(9988):46-55. doi: 10.1016/S0140-6736(15)60690-0 [published Online First: 2015/04/22]
22. Jolly K, Taylor R, Lip GY, et al. The Birmingham Rehabilitation Uptake Maximisation Study (BRUM). Home-based compared with hospital-based cardiac rehabilitation in a multi-ethnic population: cost-effectiveness and patient adherence. *Health Technol Assess* 2007;11(35):1-118. [published Online First: 2007/09/05]
23. Lamb SE, Williams MA, Williamson EM, et al. Managing Injuries of the Neck Trial (MINT): a randomised controlled trial of treatments for whiplash injuries. *Health Technol Assess* 2012;16(49):iii-iv, 1-141. doi: 10.3310/hta16490 [published Online First: 2012/12/18]
24. Logan PA, Armstrong S, Avery TJ, et al. Rehabilitation aimed at improving outdoor mobility for people after stroke: a multicentre randomised controlled study (the Getting out of the House Study). *Health Technol Assess* 2014;18(29):vii-viii, 1-113. doi: 10.3310/hta18290 [published Online First: 2014/05/09]
25. McCarthy CJ, Mills PM, Pullen R, et al. Supplementation of a home-based exercise programme with a class-based programme for people with osteoarthritis of the knees: a randomised controlled trial and health economic analysis. *Health Technol Assess* 2004;8(46):iii-iv, 1-61. [published Online First: 2004/11/06]
26. Sackley CM, Walker MF, Burton CR, et al. An Occupational Therapy intervention for residents with stroke-related disabilities in UK Care Homes (OTCH): cluster randomised controlled trial with economic evaluation. *Health Technol Assess* 2016;20(15):1-138. doi: 10.3310/hta20150 [published Online First: 2016/03/02]
27. Underwood M, Lamb SE, Eldridge S, et al. Exercise for depression in care home residents: a randomised controlled trial with cost-effectiveness analysis (OPERA). *Health Technol Assess* 2013;17(18):1-281. doi: 10.3310/hta17180 [published Online First: 2013/05/02]
28. Vickers AJ, Rees RW, Zollman CE, et al. Acupuncture of chronic headache disorders in primary care: randomised controlled trial and economic analysis. *Health Technol Assess* 2004;8(48):iii, 1-35. [published Online First: 2004/11/06]
29. Waterhouse JC, Walters SJ, Oluboyede Y, et al. A randomised 2 x 2 trial of community versus hospital pulmonary rehabilitation, followed by telephone or conventional follow-up. *Health*

For peer review only - http://bmjopen.bmj.com/site/about/guidelines.xhtml

Technol Assess 2010;14(6):i-v, vii-xi, 1-140. doi: 10.3310/hta14060 [published Online First: 2010/02/12]

30. Weindling AM, Cunningham CC, Glenn SM, et al. Additional therapy for young children with spastic cerebral palsy: a randomised controlled trial. *Health Technol Assess* 2007;11(16):iii-iv, ix-x, 1-71. [published Online First: 2007/04/28]
31. Williams MA, Williamson EM, Heine PJ, et al. Strengthening And stretching for Rheumatoid Arthritis of the Hand (SARAH). A randomised controlled trial and economic evaluation. *Health Technol Assess* 2015;19(19):1-222. doi: 10.3310/hta19190 [published Online First: 2015/03/10]
32. Underwood M, Eldridge S, Lamb S, et al. The OPERA trial: protocol for a randomised trial of an exercise intervention for older people in residential and nursing accommodation. *Trials* 2011;12:27. doi: 10.1186/1745-6215-12-27
33. Moher D, Schulz KF, Altman DG. The CONSORT statement: revised recommendations for improving the quality of reports of parallel group randomised trials. *Lancet* 2001;357:1191-94.
34. Boote J, Baird W, Sutton A. Public involvement in the systematic review process in health and social care: a narrative review of case examples. *Health Policy* 2011;102(2-3):105-16. doi: 10.1016/j.healthpol.2011.05.002 [published Online First: 2011/06/07]
35. O'Cathain A, Croot L, Sworn K, et al. Taxonomy of approaches to developing interventions to improve health: a systematic methods overview. *Pilot Feasibility Stud* 2019;5:41. doi: 10.1186/s40814-019-0425-6 [published Online First: 2019/03/30]
36. Arrowsmith J. Trial watch: Phase II failures: 2008-2010. *Nat Rev Drug Discov* 2011;10(5):328-9. doi: 10.1038/nrd3439 [published Online First: 2011/05/03]
37. Petticrew M, Rehfuess E, Noyes J, et al. Synthesizing evidence on complex interventions: how meta-analytical, qualitative, and mixed-method approaches can contribute. *J Clin Epidemiol* 2013;66(11):1230-43. doi: 10.1016/j.jclinepi.2013.06.005 [published Online First: 2013/08/21]
38. Djulbegovic B. Acknowledgment of uncertainty: a fundamental means to ensure scientific and ethical validity in clinical research. *Curr Oncol Rep* 2001;3(5):389-95. [published Online First: 2001/08/08]
39. Angst F, Aeschlimann A, Stucki G. Smallest detectable and minimal clinically important differences of rehabilitation intervention with their implications for required sample sizes using WOMAC and SF-36 quality of life measurement instruments in patients with osteoarthritis of the lower extremities. *Arthritis and rheumatism* 2001;45(4):384-91. doi: 10.1002/1529-0131(200108)45:4<384::AID-ART352>3.0.CO;2-0 [published Online First: 2001/08/15]
40. De Meulemeester J, Fedyk M, Jurkovic L, et al. Many randomized clinical trials may not be justified: a cross-sectional analysis of the ethics and science of randomized clinical trials. *J Clin Epidemiol* 2018;97:20-25. doi: 10.1016/j.jclinepi.2017.12.027 [published Online First: 2018/01/07]
41. Bleijenberg N, de Man-van Ginkel JM, Trappenburg JCA, et al. Increasing value and reducing waste by optimizing the development of complex interventions: Enriching the development phase of the Medical Research Council (MRC) Framework. *International journal of nursing studies* 2018;79:86-93. doi: 10.1016/j.ijnurstu.2017.12.001 [published Online First: 2017/12/09]

For peer review only

For peer review only - http://bmjopen.bmj.com/site/about/guidelines.xhtml

Figure Legends:

Figure 1 Study selection

Figure 2 Classification of Primary Outcome

Figure 3 Treatment success of included trials based on 95% Confidence Intervals and Minimum Clinically Important Difference from sample size calculation (d)

For peer review only

For peer review only - http://bmjopen.bmj.com/site/about/guidelines.xhtml

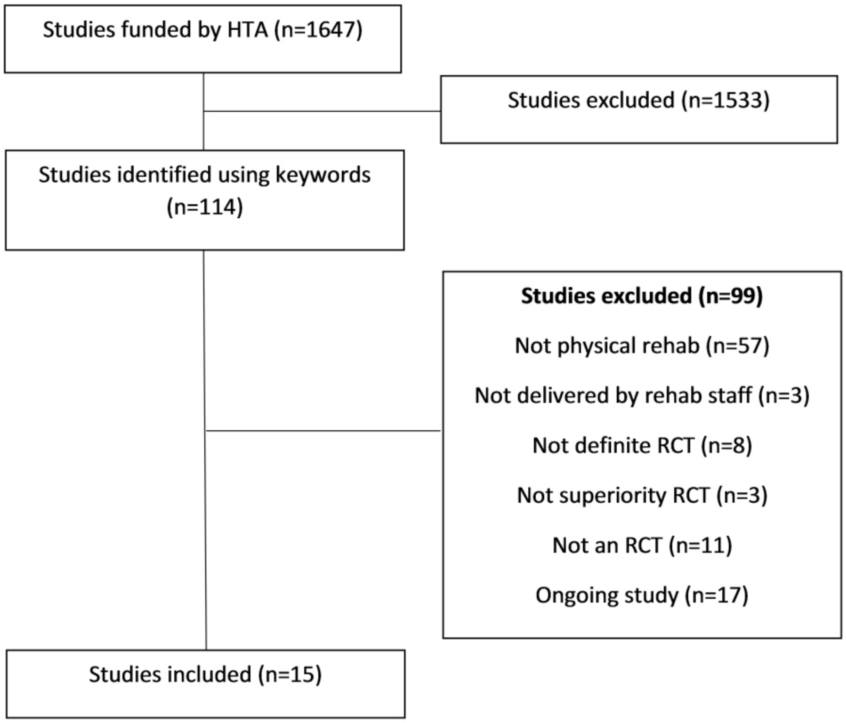

Figure 1 Study selection

Figure 1 Study selection

89x82mm (300 x 300 DPI)

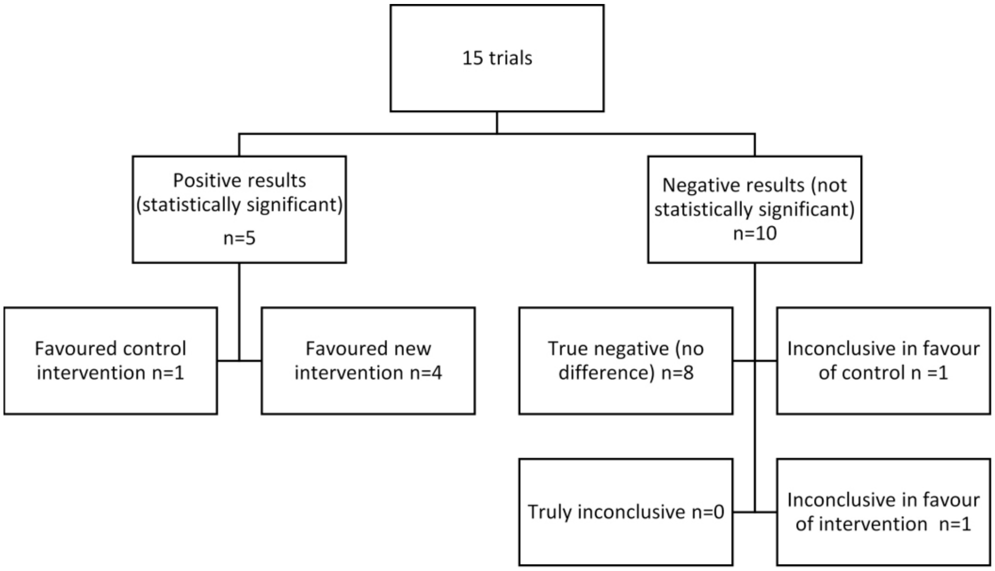

Figure 2 Classification of primary outcome

Figure 2 Classification of Primary Outcome

89x66mm (300 x 300 DPI)

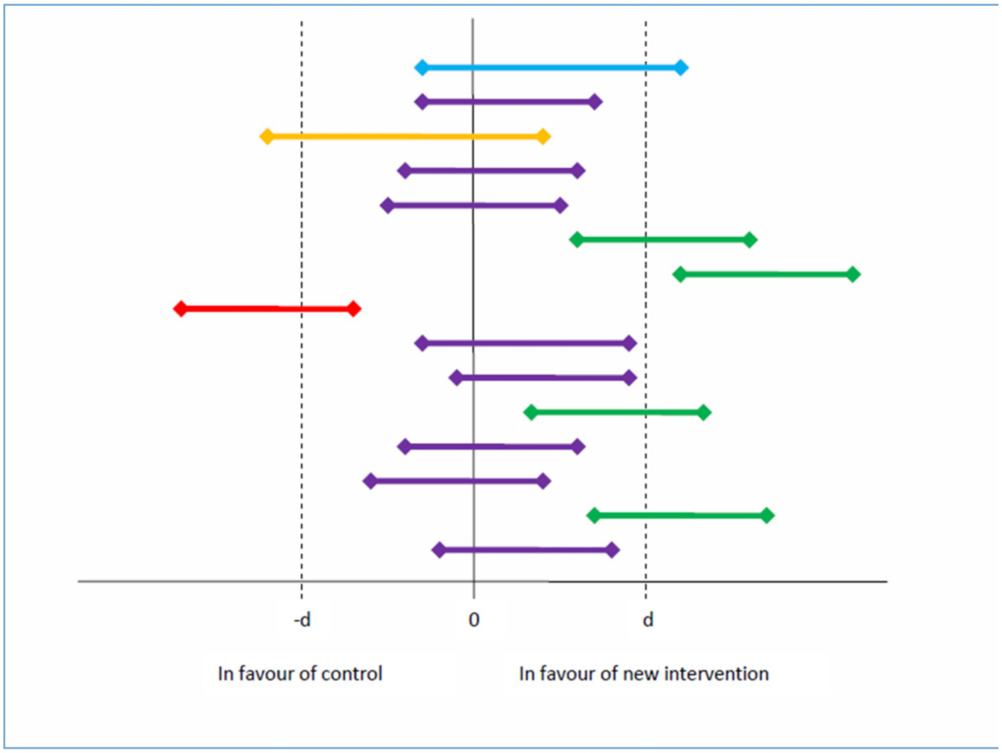

Figure 3 Treatment success of included trials based on 95% Confidence Interval and Minimal Clinically Important Difference from trial sample size calculation (d)

Key: *Green = statistically significantly in favour of intervention; Red = statistically significantly in favour of control; Blue = Inconclusive in favour of intervention; Yellow = Inconclusive in favour of control; Purple = True negative (no difference)*

Figure 3 Treatment success of included trials based on 95% Confidence Interval and Minimal Clinically Important Difference from trial sample size calculation (d)

87x89mm (300 x 300 DPI)

BMJ Open

Intervention development and treatment success in UK Health Technology Assessment funded trials of physical rehabilitation: a mixed methods analysis

Journal:	*BMJ Open*
Manuscript ID	bmjopen-2018-026289.R3
Article Type:	Research
Date Submitted by the Author:	24-Jul-2019
Complete List of Authors:	Goodwin, Victoria; University of Exeter Medical School<br>Hill, Jacqueline; University of Exeter Medical School<br>Fullam, James; University of Exeter Medical School<br>Finning, Katie; University of Exeter Medical School<br>Pentecost, C; University of Exeter Medical School<br>Richards, David; University of Exeter, Medical School
**Primary Subject Heading**:	Health services research
Secondary Subject Heading:	Rehabilitation medicine
Keywords:	rehabilitation, intervention development, mixed methods, randomised controlled trials

SCHOLARONE™
Manuscripts

Intervention development and treatment success in UK Health Technology Assessment funded trials of physical rehabilitation: a mixed methods analysis

*Victoria A Goodwin, University of Exeter, Exeter UK

Jacqueline J Hill, University of Exeter, Exeter UK

James Fullam, University of Exeter, Exeter UK

Katie Finning, University of Exeter, Exeter UK

Claire Pentecost, University of Exeter, Exeter UK

David A Richards, University of Exeter, Exeter UK

* Corresponding author: 2.26 South Cloisters, University of Exeter Medical School, St Luke's Campus, Magdalen Road, Exeter EX1 2LU. Telephone 01392 722745 v.goodwin@exeter.ac.uk

For peer review only

Keywords

Rehabilitation, randomised controlled trials, quality, intervention development, mixed methods

For peer review only - http://bmjopen.bmj.com/site/about/guidelines.xhtml

Abstract

Objectives: Physical rehabilitation is a complex process and trials of rehabilitation interventions are increasing in number but often report null results. This study aimed to establish treatment success rates in physical rehabilitation trials funded by the National Institute of Health Research Health Technology Assessment (NIHR HTA) programme and examine any relationship between treatment success and the quality of intervention development work undertaken.

Design: Mixed methods study

Setting: UK

Methods:

The NIHR HTA portfolio was searched for all completed definitive randomised controlled trials of physical rehabilitation interventions from inception to July 2016. Treatment success was categorised according to criteria developed by Djulbegovic and colleagues. Detailed textual data regarding any intervention development work were extracted from trial reports and supporting publications and informed the development of quality ratings. Mixed methods integrative analysis was undertaken to explore the relationship between quantitative and qualitative data using joint displays.

Results: Fifteen trials were included in the review. Five reported a definitive finding, four of which were in favour of the 'new' intervention. Eight trials reported a true negative (no difference) outcome. Integrative analysis indicated those with lower quality intervention development work were less likely to report treatment success.

Conclusions: Despite much effort and funding, most physical rehabilitation trials report equivocal findings. Greater focus on high quality intervention development may reduce the likelihood of a null result in the definitive trial, alongside high quality trial methods and conduct.

Strengths and limitations of this study:

- To our knowledge, this study is the first to use mixed methods integrative analyses to explore the relationship between quality of intervention development work and treatment success.
- Using the NIHR HTA Journal monographs, published protocols and other supporting publications for each study together provided a detailed and rich source of data beyond what would be found in a single traditional journal publication.
- The study reviewed randomised controlled trials of physical rehabilitation from a single UK funder as an exemplar and therefore findings may not be representative of other complex interventions or other funding bodies.

For peer review only

BACKGROUND

Rehabilitation is *"a set of interventions designed to optimise function and reduce disability in individuals with health conditions in interaction with their environment".*[1] and is an essential aspect of healthcare provision. By its very nature rehabilitation in clinical practice is an individually focused, complex activity, involving interventions that are multi-faceted and often implicit in nature[2] and as such, historically, this has been viewed as a barrier to undertaking research.[3] This said, there is a growing body of randomised controlled trials (RCTs) of rehabilitation, suggesting that these challenges can be overcome.[4] This may, in part, be supported by the publication of the Medical Research Council (MRC) Framework for developing and evaluating complex interventions.[5 6]

The MRC framework was developed to optimise the likelihood that new interventions are not rejected as being ineffective when inadequate effort has been made in the development of the intervention.[7] Likewise, Chalmers and Glasziou[8] highlighted the importance of avoiding research waste and recommended that sufficient effort is made to ensure the relevant research questions are identified and addressed using high quality research methods. However, there appears to have been no formal evaluation of the impact of using the development component of the framework on trial outcomes and whether we are observing evidence of effective interventions being developed.

Previous UK[9] and USA[10] reviews synthesised successful and non-successful treatment outcomes from trials of new interventions in order to assess the equipoise principle and to understand what return has been achieved on the investment made by those taking part in the trials, researchers and funders. Dent and Raftery[9] reported 24% (20/85) primary outcome comparisons as having a positive result, of which 16/85 (19%) were in favour of the new intervention, with 19/85 (22%) comparisons reporting a true negative outcome. However, these authors did not focus on rehabilitation interventions, nor did they seek to understand factors that may impact on treatment success, such as the quality or intensity of intervention development pre-trial procedures. Informal discussions with colleagues in the UK and internationally noted that an increasing number of publically funded, large RCTs evaluating physical rehabilitation interventions had reported null findings. Similar concerns have been reported in studies of public health interventions.[11 12] Our study, therefore, sought to assess this observation and also explore whether intervention development activities contributed to treatment success using the National Institute of Health Research Health Technology Assessment programme (NIHR HTA) as an exemplar.

We aimed to use data from the NIHR HTA to:

(a) Establish the treatment outcomes of funded RCTs of physical rehabilitation;

(b) Establish how many new interventions were found to be effective;

(c) Examine what work had been done in terms of developing the new intervention;

(d) Examine the relationship between (a) and (c).

We adopted a mixed methods approach to address the study aims. Although evidence of using integrative mixed methods approaches in synthesising evidence on complex interventions is limited, mixing together qualitative and quantitative data can generate understanding that has the potential to be greater than the sum of the individual parts.[13]

For peer review only - http://bmjopen.bmj.com/site/about/guidelines.xhtml

METHODS

Design

We undertook a review of NIHR HTA funded randomised controlled trials of physical rehabilitation interventions using narrative synthesis of outcomes and mixed methods analysis of the relationship between intervention development and categorical treatment outcomes using joint displays.

Patient and Public Involvement

Patients and the public were not involved in this study.

Data sources and inclusion criteria

We included superiority randomised controlled trials of physical rehabilitation funded by the NIHR HTA programme. The interventions could be delivered by a single profession or be multi-professional. The NIHR HTA programme is the leading public funding source for randomised controlled trials (RCTs) in the UK and trials of rehabilitation are increasingly part of the portfolio. We only included completed RCTs whose main trial findings were reported in an HTA monograph or peer-reviewed publication in order to establish treatment success. We excluded: pilot and feasibility RCTs as they do not aim to assess the efficacy or effectiveness of interventions;[14] studies where the interventions were primarily psychological or cognitive as the focus of the study was physical rehabilitation; where the primary outcome findings were not reported with a 95% confidence interval (CI) as these data were required to assess treatment success.

Search and screening

We searched the HTA Project Portfolio (since superseded by the NIHR Journals Library https://www.journalslibrary.nihr.ac.uk/#/) from inception to July 2016 using the following keywords: physiotherap*OR occupational therap* OR speech and language therap* OR rehabilitation. We removed duplicates and then titles and scientific abstracts were reviewed for potential inclusion by one person and checked by a second. Subsequently full text reports were screened for inclusion by one person and checked by a second. Any disagreements were discussed and agreed with a third person.

Data extraction

All data were extracted by one person and checked by a second. Discrepancies were discussed and resolved with a third person.

Quantitative Trial data: Data extracted from each trial publication included trial design, target population, health categories (using the Health Research Classification System), primary outcome(s) and time point, minimal important clinical difference (MCID) or percentage change that the trial aimed to detect, planned and achieved sample size, and primary outcome results with 95% CI. We also recorded the professional background of the Chief Investigator and amount of funding awarded.

Qualitative Intervention development data: Using the revised version of Criteria for Reporting the Development and Evaluation of Complex Interventions (CReDECI 2)[15] and the Template for Intervention Description and Replication checklist (TIDieR)[16] as frameworks we extracted all available

For peer review only - http://bmjopen.bmj.com/site/about/guidelines.xhtml

documentary (qualitative) data from the body of the text regarding intervention development, including descriptions of underlying theory, intervention components and reasons for selection, intended interactions between components, contextual considerations, piloting of intervention and impact of definitive intervention to be evaluated, control components, planned intervention delivery and materials. Where additional supporting publications were cited, such as a protocol or intervention development studies, we used these as additional sources of documentary data.

Data analysis

We used summary statistics to describe the characteristics of the included studies. We categorised primary outcome findings into one of six treatment outcome categories as described by Djulbegovic and colleagues,[10] these being: 1) statistically significant in favour of the new treatment, 2) statistically significant in favour of the control treatment 3) true negative, 4) truly inconclusive, 5) inconclusive in favour of new treatment or 6) inconclusive in favour of the control treatment. This was achieved by comparing the 95% confidence interval for the difference in primary outcome to the difference specified in the sample size calculation.[9] If the 95% confidence interval excluded a meaningful difference in either direction, implying the treatments have similar effects, the results were categorised as true negative. If the 95% confidence interval included a meaningful difference in either direction (i.e. trial failed to answer the primary question), the results were categorised as being truly inconclusive.

Where a single primary outcome and primary time point were not explicitly identified we utilised the following hierarchy to determine which primary outcome would be used in the analysis:

- Explicitly defined primary outcome
- Outcome used in power calculation
- Main outcome stated in trial objectives
- First outcome reported in sample size calculation

If a primary time point was not reported we used the first follow up time point as this is when we would expect the intervention to have had the greatest effect.

Our preliminary analysis of the qualitative documentary data involved the reading and re-reading of source documents and the extracted descriptions to consolidate our understanding of the development work undertaken in each study. Using a reflective and iterative process we undertook thematic analysis to distil, structure and make sense of intervention development activity by coding and organising data into themes and subthemes. Each theme and sub-theme provided a coherent description of the development work undertaken for each study, which were then synthesised into short descriptors to allow us to produce summary tables. The summary tables comprised a row for each study with columns for each theme and, where relevant, each subtheme. A second researcher checked, discussed and refined descriptors to ensure accuracy. From these descriptions we then developed descriptive ratings on the quality of the intervention development. Depending on the nature of the data, ratings were categorised and the iterative process involved two researchers refining and checking ratings to ensure they reflected the summary data from each study. In order to provide a visual representation of the quality of intervention development work these ratings were then converted to a quality coding to indicate high quality, some or unclear quality or limited quality. For example under co-design the highest quality rating was given when the intervention was co-

For peer review only - http://bmjopen.bmj.com/site/about/guidelines.xhtml

For peer review only

designed with *both* clinical and service user input, a middle rating when *either* clinicians or service users were involved, and the lowest quality rating when *neither* clinicians nor service users were involved.

To examine the relationship between intervention development and treatment success, we applied mixed methods analytical techniques in novel ways. For each study, we combined ratings derived from the qualitative data on intervention development with the quantitative data on treatment outcomes in a joint display.

RESULTS

We included fifteen RCTs (Figure 1),[17-31] of which thirteen used a two-arm, parallel RCT design, one was a two-arm cluster RCT and one was a four-arm factorial design (of which only two arms related to physical rehabilitation). Table 1 provides a summary of the population, intervention, control and outcomes for each study. The combined sample size required to demonstrate a true difference in primary outcomes (excluding any inflation to account for loss to follow up) was 7548 participants. The total number of participants who provided primary outcome data was higher than this (n=7834), likely due to lower loss to follow up that estimated, although three studies[19 29 30] were considerably below their target sample size at the primary time point. Five primary outcomes were symptom-based or clinical outcomes, seven were functional measures, two were combined measures and one assessed quality of life. Primary time points varied from immediately post-intervention to one year (median 6 months). The health categories were: Stroke (n=4), Neurological conditions (n=2), Inflammatory/Immune system disorders (n=2), Respiratory (n=1), Musculoskeletal (n=1), Cardiovascular (n=1), Mental Health (n=1), Accident/injuries (n=1), Renal/urogenital (n=1) and other (n=1). Seven interventions were delivered by physiotherapists, one by occupational therapists, one by speech and language therapists, one by nurses, two could be delivered by either a physiotherapist or a nurse, two could be delivered by a physiotherapist or an occupational therapist and one was delivered by both a physiotherapist and an occupational therapist. The Chief Investigators leading the studies were physicians (n=7), physiotherapists (n=5), occupational therapists (n=1), psychologists (n=1) and methodologists (n=1). The total amount of research funding awarded was £12,515,823.

One third of studies (5/15) reported a definitive finding in favour of one of the treatment arms - four studies in favour of the new treatment, one in favour of the control. Of those with negative results, eight studies reported a true negative (no difference) outcome, one was inconclusive in favour of the new treatment, and one inconclusive in favour of the control treatment (Figures 2 and 3).

Qualitative data informed two themes and ten sub-themes which enabled us to develop data-driven quality ratings:

1. *Preparatory work* (Need for the study, underpinning theory for the intervention, co-design, context considerations and intervention piloting)
2. *Intervention and control* (Intervention content and dose, individual tailoring, adherence strategies, standardised training, control content and dose)

Table 2 provides examples of summary data underpinning each rating with Table 3 describing the quality rating for each study in chronological order. Table 4 presents the integrative qualitative and

For peer review only - http://bmjopen.bmj.com/site/about/guidelines.xhtml

quantitative analysis using a joint display. No single study was deemed to be high quality in each sub-theme. This said, the two best rated studies reported only expert clinical input into co-designing the intervention with a lack of clear patient and public involvement, however, they reported a definitive trial outcome in favour of the new intervention. There does not appear to be a single aspect of intervention development driving study outcomes. This said, those with lower quality development work appear more likely to show no difference in outcomes compared with those with higher quality development work. Some areas of intervention development appear to be improving with time, these being articulating a clear need and theoretical underpinning, co-design, piloting and descriptions of intervention and control components.

For peer review only

For peer review only - http://bmjopen.bmj.com/site/about/guidelines.xhtml

Table 1 Summary of included studies

Author (year published)	Funding awarded (£)	Population (target sample size/number of participants with primary outcome data)	Intervention	Control	Primary Outcome (MCID or % change study aimed to detect)
McCarthy et al (2004)	218,517	People with knee osteoarthritis (n=152/200)	Twice weekly exercise group for 8 weeks plus home exercises	Home exercises	Aggregate Locomotor Function score (4 seconds)
Vickers et al (2004)	161,532	People with chronic headache (n=288/301)	Up to 12 acupuncture treatments plus usual care	Usual care from General Practitioner	Weekly headache score (35% reduction)
Epps et al (2005)	152,011	Children with juvenile arthritis (n=200/74)	8 hydrotherapy and 8 land based sessions over 2 weeks followed by weekly/fortnightly hydrotherapy for 2 months	16 land based exercise sessions over 2 weeks followed by weekly or fortnightly land based exercise sessions	Disease status calculated from Childhood Health Assessment Questionnaire (CHAQ), physicians' global assessment of disease activity, parents' global assessment of overall well-being, number of joints with limited ROM, number of active joints, erythrocyte sedimentation rate (30% improvement on 3 measures with < 30% deterioration on remaining 3 measures)
Weindling et al (2007)	334,093	Children with cerebral palsy (n=153/76)	Regular physiotherapy (usual care) plus additional weekly session from physiotherapy assistant for 6 months	Usual care (regular physiotherapy)	Gross Motor Function Measure (14 points)
Jolly et al (2007)	480,612	People with myocardial infarction or revascularisation (n=450/487)	Home-based self-help manual plus up to 3 face to face and 1 phone call support over 12 weeks	Centre-based cardiac rehabilitation	Incremental shuttle walk test (6 shuttles); Hospital anxiety and depression scale (1.5 points); smoking cessation (20%); blood pressure (6mmHg systolic); serum cholesterol (0.4 mmol/l)
Waterhouse et al (2010)	460,543	People with Chronic Obstructive Pulmonary Disease (n=372/162)	Twice weekly community-based pulmonary rehabilitation	Twice weekly hospital-based pulmonary rehabilitation	Endurance Shuttle Walk Test (60% increase in distance walked)
Glazener et al (2011)	1,051,699	Men with incontinence post-prostate surgery (696/788)	Assessment and treatment and exercise over 4 face to face sessions plus advice leaflet	Advice leaflet	Self-reported urinary incontinence (15% reduction in % of people with urinary incontinence)
Bowen et al (2012)	1,457,533	Adults with aphasia or dysarthria after stroke (n=170/153)	Speech and language therapy visits up to 3 sessions per week for up to 16 weeks	Volunteer visits up to 3 sessions per week for up to 16 weeks	Therapy Outcome measure (0.5)
Lamb et al (2012)	755,310	People with whiplash with persistent symptoms (n=422/507)	6 sessions of assessment and treatment/exercise over 8 weeks	Single session of advice	Neck Disability Index (3 points)
Underwood et al (2013)	1,957,884	Care home residents (n=409/493)	Twice weekly exercise group for a year	Depression awareness training for care home staff	Geriatric Depression Scale (17.3% reduction in % of people with depression)
Logan et al (2014)	993,080	People with stroke (n=440/503)	Up to 12 therapy visits to increase outdoor mobility plus verbal/written advice	Verbal/written advice	SF-36 Social function domain (12.5 points)
Williams et al (2015)	976,955	People with rheumatoid arthritis (n=352/438)	6 sessions of exercise plus home exercises over 12 weeks	Single assessment advice session with 2 further optional sessions over 12 weeks (no exercises)	Michigan Hand Outcome Questionnaire (0.3)
AVERT Group (2015)	282,372	People with stroke (n=2104/2083)	3 additional out of bed sessions per day for up to 2 weeks	Usual care	Modified Rankin Scale-(mRS) (7.1% Absolute risk reduction of a mRS score of 3-6)

For peer review only

Sackley et al (2016)	1,797,676	Care home residents with stroke (n=660/870)	Individualised occupational therapy	No occupational therapy	Barthel Index (2 points)
Clarke et al (2016)	1,436,006	People with Parkinson's (n=680/699)	Up to 8 individualised sessions of Physiotherapy and up to 8 individualised sessions of occupational therapy	No therapy	Nottingham Extended Activities of Daily Living (2.5 points)

For peer review only

For peer review only - http://bmjopen.bmj.com/site/about/guidelines.xhtml

Table 2 Description of themes, subthemes and quality ratings with examples

Theme	Sub-theme	Description of rating	Examples of data supporting rating	Rating
**Preparatory work**	**Need for the study**	Multiple sources of evidence of need for the study e.g. recent systematic review, guidelines, high level reports, commissioned research, national audit	International task force highlighted lack of evidence and need for evaluation. Cochrane review drew similar conclusions.	▲
		Single source of evidence / non-systematic review to support need for study	Old systematic review indicates paucity of high quality research.	■
		Lack of clarity or underpinning evidence regarding need for study	Poor justification for the study. Evidence cited doesn't support the need for this particular study.	●
	**Theoretical underpinning**	Theoretical underpinning described	Physiological and psychological theories underpinning the intervention described in detail.	▲
		Lacks clear theoretical underpinning	No information provided regarding the theoretical basis for the intervention provided.	■
	**Co-design**	Good PPI and expert clinical input	Patients and clinicians helped develop the intervention.	▲
		Good PPI but weak or no expert clinical input / Good clinical input but unclear or no PPI	Clinicians contributed to the intervention development but no indication of service user involvement.	■
		No co-design	No co-design was undertaken to develop the intervention.	●
	**Contextual considerations**	Context considered	The use of different professionals in delivering the intervention reflected the real world situation of how this would occur in practice.	▲
		Context not adequately considered	There was a lack of understanding of relevant context and factors needed for intervention development and delivery.	●
	**Piloting of intervention**	Pilot conducted, evaluated and findings addressed for main evaluation	The pilot data helped refine the intervention for evaluation in the main trial.	▲
		Pilot conducted but findings not clearly addressed in intervention for main evaluation	The pilot work led to a modification of the control intervention but unclear as to whether this also happened for the novel intervention.	■
		No pilot reported	No piloting of intervention reported	●

For peer review only

For peer review only - http://bmjopen.bmj.com/site/about/guidelines.xhtml

**Intervention and control**	**Content and dose**	Intervention components and dose clearly described	The content and the dose of the exercise programme was described in detail.	▲
		Intervention components clearly described but dose was not standardised	The content of the programme was well described but no specific dose was prescribed.	■
		Intervention not replicable from description of components and dose	Intervention was based on usual practice and had no protocol or guidance on minimum dose.	●
	**Tailoring**	Formalised assessment to inform tailoring	An assessment tool was used to determine the individuals level of exercise intensity	▲
		Clinical judgement only used to inform tailoring	Therapists used their clinical judgement to individually tailor programmes.	■
		Not adequately reported	Intervention individually tailored but no information as to how this was undertaken.	●
	**Adherence support strategies**	Explicit strategies to support adherence to the intervention clearly reported	Specific adherence strategies described as part of the intervention.	▲
		No clear information regarding adherence support strategies	No information reported regarding adherence strategies.	●
		Supporting adherence is not relevant to the intervention	The intervention was passive and adherence strategies not relevant.	NA
	**Intervention training**	Standardised training in intervention received +/- additional/ongoing support or training	Staff attended a 1.5 day training session and had an additional support session with ongoing contact from research team.	▲
		No standardised intervention training received but staff delivering described to be experienced in the intervention or training of staff unclear/not reported	Staff have post graduate training in the intervention but no study specific training reported.	■
	**Control description**	Active control/attention control/usual care with some standardised components	Control was an active intervention that differed from intervention only in terms of delivery setting.	▲
		Usual care had no standardised components	Control was usual care and was not standardised between sites.	■

Key: ▲ High quality ■ Some/Unclear quality ● Limited quality

For peer review only

For peer review only - http://bmjopen.bmj.com/site/about/guidelines.xhtml

Table 3 Quality of intervention development work ordered by year of publication

Author (year)	Need	Theory	Co-design	Context	Pilot	Intervention Content	Tailored	Adherence strategies	Training delivery	Control description
McCarthy (2004)[25]	■	■	●	▲	●	▲	▲	■	■	▲
Vickers (2004)[28]	■	■	●	▲	●	●	●	NA	■	■
Epps (2005)[19]	■	▲	■	●	■	▲	■	■	■	▲
Weindling (2007)[30]	●	▲	●	●	●	●	●	■	■	■
Jolly (2007)[22]	●	▲	●	▲	●	▲	■	▲	▲	■
Waterhouse (2010)[29]	▲	■	●	●	●	▲	■	▲	■	▲
Glazener (2011)[20]	▲	▲	▲	▲	●	▲	▲	▲	▲	▲
Bowen (2012)[17]	▲	▲	■	▲	▲	■	▲	▲	■	▲
Lamb (2012)[23]	▲	▲	■	▲	▲	▲	▲	▲	▲	▲
Underwood (2013)[32]	▲	▲	■	▲	▲	▲	▲	▲	▲	▲
Logan (2014)[24]	▲	▲	▲	▲	■	■	▲	▲	▲	▲
AVERT Group (2015)[21]	▲	▲	●	▲	■	▲	▲	NA	▲	■
Williams (2015)[31]	▲	▲	■	▲	▲	▲	▲	▲	▲	▲
Sackley (2016)[26]	▲	▲	■	▲	■	●	▲	▲	■	■
Clarke (2016)[18]	●	■	■	▲	●	●	▲	■	■	■

Key:

▲ High quality ■ Some/Unclear quality ● Limited quality

For peer review only

Table 4 Joint display of treatment success ordered by quality of intervention development work

Author	Need	Theory	Co-design	Context	Pilot	Intervention Content	Tailored	Adherence strategies	Training delivery	Control description	Treatment success
Lamb[23]	▲	▲	■	▲	▲	▲	▲	▲	▲	▲	Statistically significant in favour of intervention
Williams[31]	▲	▲	■	▲	▲	▲	▲	▲	▲	▲	Statistically significant in favour of intervention
Underwood[32]	▲	▲	■	▲	▲	▲	▲	▲	▲	▲	True negative (No difference)
Glazener[20]	▲	▲	▲	▲	●	▲	▲	▲	▲	▲	True negative (No difference)
Logan[24]	▲	▲	▲	▲	■	■	▲	▲	▲	▲	True negative (No difference)
Bowen[17]	▲	▲	■	▲	▲	■	▲	▲	■	▲	Inconclusive in favour of intervention
AVERT Group[21]	▲	▲	●	▲	■	▲	▲	NA	▲	■	Statistically significant in favour of control
Sackley[26]	▲	▲	■	▲	■	●	▲	▲	■	■	True negative (No difference)
Jolly[22]	●	▲	●	▲	●	▲	■	▲	▲	■	True negative (No difference)
McCarthy[25]	■	■	●	▲	●	▲	▲	■	■	▲	Statistically significant in favour of intervention
Waterhouse[29]	▲	■	●	●	●	▲	■	▲	■	▲	True negative (No difference)
Epps[19]	■	▲	■	●	■	▲	■	■	■	▲	Inconclusive in favour of control
Clarke[18]	●	■	■	▲	●	●	▲	■	■	■	True negative (No difference)
Vickers[28]	■	■	●	▲	●	●	●	NA	■	■	Statistically significant in favour of intervention
Weindling[30]	●	▲	●	●	●	●	●	■	■	■	True negative (No difference)

Key:

▲ High quality ■ Some/Unclear quality ● Limited quality

For peer review only

DISCUSSION

Physical rehabilitation research targets a broad population although we found that studies for people with stroke to be the most common (n=4). We established that only one third (5/15) of the randomised controlled trials of physical rehabilitation funded by the NIHR HTA programme successfully demonstrated a statistically significant effect for one of the randomised groups in each trial. Four (27%) trials found an effect in favour of the 'new' intervention. Although we would not expect all studies to demonstrate effectiveness in favour of the 'new' intervention, the equipoise principle implies that there would be no difference between the proportion of studies favouring intervention or control[9]. However, this doesn't account for a null outcome. We were able to use contemporary research methods to develop an assessment of the quality of development work and assessed the included trials to be of varied quality in terms of intervention development work. In general, we found that comprehensive intervention development may have a positive relationship with treatment success. Two studies[23 31] with high quality intervention development reported treatment success although two older[25 28] and possibly less well reported trials also reported effective interventions. Developments in complex intervention evaluation[5], reporting standards[16 33] and involving patients and the public in research[34] have occurred since the inception of the HTA programme and as such some development work may have been undertaken but not reported in the older studies. A recent overview of approaches to developing interventions noted the absence of patient and public involvement[35]. In addition, there was limited evidence of piloting the intervention prior to proceeding to the full trial with only four studies reporting this having been done. Most (> 80%) drug intervention development studies fail to reach the 'Phase III' trial stage.[36] Public health interventions have tended to go straight to an RCT without piloting which may contribute to challenges in demonstrating effectiveness.[11] There are of course other factors that influence trial findings, including trial methods and conduct, however our question was specifically determined to explore what, if any, relationship existed between intervention development and outcomes and not in the effectiveness of particular interventions.

A strength of our study is the use of integrative mixed methods analysis which has enabled us to explore the relationship between development work and outcome. This rarely used approach in evidence synthesis[37] has given us a unique insight that would not have been possible using a quantitative or qualitative analysis alone. A limitation of our work could be the focus on a single UK funding stream which does not necessarily reflect the body of research funded from other sources and therefore the quality of intervention development work is not necessarily generalizable. However, the NIHR HTA programme is the single largest funder of randomised controlled trials of applied health research in the UK. They publish detailed monographs of their funded studies, along with protocols and other supporting publications that provide a detailed and rich source of data beyond what would normally be available in journal-based peer reviewed publications alone. We were able to retain the essence and nuances of the qualitative data whilst developing categorical ratings of quality to help us better explore the relationship between development work and treatment success.

Our findings are similar to those of Dent and Raftery[9] in relation to those trials showing a benefit who reported 19% (16/85) of studies found in favour of the new intervention. It has been suggested that a 50% success rate is a good investment for healthcare research,[38] however, our findings indicate that the studies we reviewed fell well below this. In contrast, we observed a considerably larger proportion of true negative studies (8/15; 53%) compared with 19/85 (22%) reported by Dent and Raftery.[9] The

For peer review only - http://bmjopen.bmj.com/site/about/guidelines.xhtml

difference is even greater when compared with a review of cancer trials in the USA where only 2% of trials found a true negative outcome.[10] The reasons for the differences are unclear but could include the pragmatic nature of HTA funded trials and the relative smaller effect sizes often associated with trials of rehabilitation.[39]

It has been recently suggested that RCTs should only be undertaken if they are justified both scientifically and ethically by having a clear hypothesis and established uncertainty[40] and our findings support that by way of good quality intervention development work. Our findings also align with the elements suggested to be key for developing interventions and reducing research waste by increasing the likelihood of success[41] which will form a comprehensive supplement to the development phase of the updated MRC guidance on developing and evaluating interventions due for publication in 2019. The NIHR HTA is publically funded and by increasing effort and focus on developing rehabilitation and other interventions in the future researchers and funding bodies could increase the possibility of a definitive trial reporting beneficial findings after much investment of time and public money.

CONCLUSIONS

Despite much research effort and funding, only four out of fifteen evaluations of 'new' rehabilitation interventions funded by the NIHR HTA programme were found to be unequivocally effective. Most studies reported no difference in outcome between study arms. We have used mixed methods research to explore the relationship between intervention development work and treatment success and developed a method of assessing the quality of this work which suggests comprehensive intervention development work may have a positive relationship with treatment success.

RECOMMENDATIONS

As this was an exploratory study, further work should be undertaken to establish the validity of quality assessment of intervention development work. This said, researchers and funding agencies should not undervalue the potential benefit of high quality intervention development work prior to definitive randomised controlled trials to reduce the likelihood of a null outcome and improve current rates of treatment success.

ACKNOWLEDGEMENT

This work was supported by the NIHR Collaboration for Leadership in Applied Health Research and Care South West Peninsula (PenCLAHRC). The views expressed are those of the authors and not necessarily the NHS, the NIHR or the Department of Health and Social Care.

COMPETING INTERESTS

None

DATA SHARING STATEMENT

No additional data are available.

AUTHOR CONTRIBUTION

For peer review only

For peer review only - http://bmjopen.bmj.com/site/about/guidelines.xhtml

VG: Conception and design, data collection, analysis and interpretation, drafting and approving the manuscript;

JH: Design, data collection, analysis and interpretation, drafting and approving the manuscript;

JF: Data collection, analysis, revising and approving the manuscript;

KF: Data collection, revising and approving the manuscript;

CP: Data collection, revising and approving the manuscript;

DR: Conception, revising and approving the manuscript.

REFERENCES

1. World Health Organization. Rehabilitation in health systems. Geneva, 2017.
2. De Souza L. Theories about therapies are underdeveloped. *Physiotherapy Research International* 1998;3(3):iv-vi.
3. Hislop HJ. Tenth Mary McMillan lecture. The not-so-impossible dream. *Phys Ther* 1975;55(10):1069-80. [published Online First: 1975/10/01]
4. Mayo N, Kaur N, Barbic S, et al. How have research questions and methods used in clinical trials published in Clinical Rehabilitation changed over the last 30 years? *Clinical Rehabilitation* 2016;30(9):847-64.
5. Medical Research Council. A framework for development and evaluation of RCT's for complex interventions to improve health. London: Medical Research Council, 2000.
6. Medical Research Council. Developing and evaluating complex interventions: new guidance. London: Medical Research Council, 2008.
7. Richards D. The complex interventions framework. In: Richards D, Hallberg I, eds. Complex interventions in health: an overview of research methods. Abingdon: Routledge 2015:5.
8. Chalmers I, Glasziou P. Avoidable waste in the production and reporting of research evidence. *Lancet* 2009;374(9683):86-9. doi: 10.1016/S0140-6736(09)60329-9 [published Online First: 2009/06/16]
9. Dent L, Raftery J. Treatment success in pragmatic randomised controlled trials: a review of trials funded by the UK Health Technology Assessment programme. *Trials* 2011;12:109. doi: 10.1186/1745-6215-12-109 [published Online First: 2011/05/06]
10. Djulbegovic B, Kumar A, Soares HP, et al. Treatment success in cancer: new cancer treatment successes identified in phase 3 randomized controlled trials conducted by the National Cancer Institute-sponsored cooperative oncology groups, 1955 to 2006. *Arch Intern Med* 2008;168(6):632-42. doi: 10.1001/archinte.168.6.632 [published Online First: 2008/03/26]
11. Hallingberg B, Turley R, Segrott J, et al. Exploratory studies to decide whether and how to proceed with full-scale evaluations of public health interventions: a systematic review of guidance. *Pilot Feasibility Stud* 2018;4:104. doi: 10.1186/s40814-018-0290-8 [published Online First: 2018/06/02]
12. Moore L, Hallingberg B, Wight D, et al. Exploratory studies to inform full-scale evaluations of complex public health interventions: the need for guidance. *J Epidemiol Community Health* 2018;72(10):865-66. doi: 10.1136/jech-2017-210414 [published Online First: 2018/07/22]
13. Barbour RS. The case for combining qualitative and quantitative approaches in health services research. *J Health Serv Res Policy* 1999;4(1):39-43. doi: 10.1177/135581969900400110 [published Online First: 1999/05/27]
14. Arain M, Campbell MJ, Cooper CL, et al. What is a pilot or feasibility study? A review of current practice and editorial policy. *BMC Medical Research Methodology* 2010;10(1):67. doi: 10.1186/1471-2288-10-67

For peer review only

For peer review only - http://bmjopen.bmj.com/site/about/guidelines.xhtml

15. Mohler R, Kopke S, Meyer G. Criteria for Reporting the Development and Evaluation of Complex Interventions in healthcare: revised guideline (CReDECI 2). *Trials* 2015;16:204. doi: 10.1186/s13063-015-0709-y [published Online First: 2015/05/04]
16. Hoffmann TC, Glasziou PP, Boutron I, et al. Better reporting of interventions: template for intervention description and replication (TIDieR) checklist and guide. *BMJ* 2014;348:g1687. doi: 10.1136/bmj.g1687 [published Online First: 2014/03/13]
17. Bowen A, Hesketh A, Patchick E, et al. Clinical effectiveness, cost-effectiveness and service users' perceptions of early, well-resourced communication therapy following a stroke: a randomised controlled trial (the ACT NoW Study). *Health Technol Assess* 2012;16(26):1-160. doi: 10.3310/hta16260 [published Online First: 2012/05/23]
18. Clarke CE, Patel S, Ives N, et al. Clinical effectiveness and cost-effectiveness of physiotherapy and occupational therapy versus no therapy in mild to moderate Parkinson's disease: a large pragmatic randomised controlled trial (PD REHAB). *Health Technol Assess* 2016;20(63):1-96. doi: 10.3310/hta20630 [published Online First: 2016/09/02]
19. Epps H, Ginnelly L, Utley M, et al. Is hydrotherapy cost-effective? A randomised controlled trial of combined hydrotherapy programmes compared with physiotherapy land techniques in children with juvenile idiopathic arthritis. *Health Technol Assess* 2005;9(39):iii-iv, ix-x, 1-59. [published Online First: 2005/09/27]
20. Glazener C, Boachie C, Buckley B, et al. Conservative treatment for urinary incontinence in Men After Prostate Surgery (MAPS): two parallel randomised controlled trials. *Health Technol Assess* 2011;15(24):1-290, iii-iv. doi: 10.3310/hta15240 [published Online First: 2011/06/07]
21. Avert Trial Collaboration group. Efficacy and safety of very early mobilisation within 24 h of stroke onset (AVERT): a randomised controlled trial. *Lancet* 2015;386(9988):46-55. doi: 10.1016/S0140-6736(15)60690-0 [published Online First: 2015/04/22]
22. Jolly K, Taylor R, Lip GY, et al. The Birmingham Rehabilitation Uptake Maximisation Study (BRUM). Home-based compared with hospital-based cardiac rehabilitation in a multi-ethnic population: cost-effectiveness and patient adherence. *Health Technol Assess* 2007;11(35):1-118. [published Online First: 2007/09/05]
23. Lamb SE, Williams MA, Williamson EM, et al. Managing Injuries of the Neck Trial (MINT): a randomised controlled trial of treatments for whiplash injuries. *Health Technol Assess* 2012;16(49):iii-iv, 1-141. doi: 10.3310/hta16490 [published Online First: 2012/12/18]
24. Logan PA, Armstrong S, Avery TJ, et al. Rehabilitation aimed at improving outdoor mobility for people after stroke: a multicentre randomised controlled study (the Getting out of the House Study). *Health Technol Assess* 2014;18(29):vii-viii, 1-113. doi: 10.3310/hta18290 [published Online First: 2014/05/09]
25. McCarthy CJ, Mills PM, Pullen R, et al. Supplementation of a home-based exercise programme with a class-based programme for people with osteoarthritis of the knees: a randomised controlled trial and health economic analysis. *Health Technol Assess* 2004;8(46):iii-iv, 1-61. [published Online First: 2004/11/06]
26. Sackley CM, Walker MF, Burton CR, et al. An Occupational Therapy intervention for residents with stroke-related disabilities in UK Care Homes (OTCH): cluster randomised controlled trial with economic evaluation. *Health Technol Assess* 2016;20(15):1-138. doi: 10.3310/hta20150 [published Online First: 2016/03/02]
27. Underwood M, Lamb SE, Eldridge S, et al. Exercise for depression in care home residents: a randomised controlled trial with cost-effectiveness analysis (OPERA). *Health Technol Assess* 2013;17(18):1-281. doi: 10.3310/hta17180 [published Online First: 2013/05/02]
28. Vickers AJ, Rees RW, Zollman CE, et al. Acupuncture of chronic headache disorders in primary care: randomised controlled trial and economic analysis. *Health Technol Assess* 2004;8(48):iii, 1-35. [published Online First: 2004/11/06]
29. Waterhouse JC, Walters SJ, Oluboyede Y, et al. A randomised 2 x 2 trial of community versus hospital pulmonary rehabilitation, followed by telephone or conventional follow-up. *Health*

For peer review only - http://bmjopen.bmj.com/site/about/guidelines.xhtml

Technol Assess 2010;14(6):i-v, vii-xi, 1-140. doi: 10.3310/hta14060 [published Online First: 2010/02/12]

30. Weindling AM, Cunningham CC, Glenn SM, et al. Additional therapy for young children with spastic cerebral palsy: a randomised controlled trial. *Health Technol Assess* 2007;11(16):iii-iv, ix-x, 1-71. [published Online First: 2007/04/28]
31. Williams MA, Williamson EM, Heine PJ, et al. Strengthening And stretching for Rheumatoid Arthritis of the Hand (SARAH). A randomised controlled trial and economic evaluation. *Health Technol Assess* 2015;19(19):1-222. doi: 10.3310/hta19190 [published Online First: 2015/03/10]
32. Underwood M, Eldridge S, Lamb S, et al. The OPERA trial: protocol for a randomised trial of an exercise intervention for older people in residential and nursing accommodation. *Trials* 2011;12:27. doi: 10.1186/1745-6215-12-27
33. Moher D, Schulz KF, Altman DG. The CONSORT statement: revised recommendations for improving the quality of reports of parallel group randomised trials. *Lancet* 2001;357:1191-94.
34. Boote J, Baird W, Sutton A. Public involvement in the systematic review process in health and social care: a narrative review of case examples. *Health Policy* 2011;102(2-3):105-16. doi: 10.1016/j.healthpol.2011.05.002 [published Online First: 2011/06/07]
35. O'Cathain A, Croot L, Sworn K, et al. Taxonomy of approaches to developing interventions to improve health: a systematic methods overview. *Pilot Feasibility Stud* 2019;5:41. doi: 10.1186/s40814-019-0425-6 [published Online First: 2019/03/30]
36. Arrowsmith J. Trial watch: Phase II failures: 2008-2010. *Nat Rev Drug Discov* 2011;10(5):328-9. doi: 10.1038/nrd3439 [published Online First: 2011/05/03]
37. Petticrew M, Rehfuess E, Noyes J, et al. Synthesizing evidence on complex interventions: how meta-analytical, qualitative, and mixed-method approaches can contribute. *J Clin Epidemiol* 2013;66(11):1230-43. doi: 10.1016/j.jclinepi.2013.06.005 [published Online First: 2013/08/21]
38. Djulbegovic B. Acknowledgment of uncertainty: a fundamental means to ensure scientific and ethical validity in clinical research. *Curr Oncol Rep* 2001;3(5):389-95. [published Online First: 2001/08/08]
39. Angst F, Aeschlimann A, Stucki G. Smallest detectable and minimal clinically important differences of rehabilitation intervention with their implications for required sample sizes using WOMAC and SF-36 quality of life measurement instruments in patients with osteoarthritis of the lower extremities. *Arthritis and rheumatism* 2001;45(4):384-91. doi: 10.1002/1529-0131(200108)45:4<384::AID-ART352>3.0.CO;2-0 [published Online First: 2001/08/15]
40. De Meulemeester J, Fedyk M, Jurkovic L, et al. Many randomized clinical trials may not be justified: a cross-sectional analysis of the ethics and science of randomized clinical trials. *J Clin Epidemiol* 2018;97:20-25. doi: 10.1016/j.jclinepi.2017.12.027 [published Online First: 2018/01/07]
41. Bleijenberg N, de Man-van Ginkel JM, Trappenburg JCA, et al. Increasing value and reducing waste by optimizing the development of complex interventions: Enriching the development phase of the Medical Research Council (MRC) Framework. *International journal of nursing studies* 2018;79:86-93. doi: 10.1016/j.ijnurstu.2017.12.001 [published Online First: 2017/12/09]

For peer review only

For peer review only - http://bmjopen.bmj.com/site/about/guidelines.xhtml

Figure Legends:

Figure 1 Study selection

Figure 2 Classification of Primary Outcome

Figure 3 Treatment success of included trials based on 95% Confidence Intervals and Minimum Clinically Important Difference from sample size calculation (d)

For peer review only

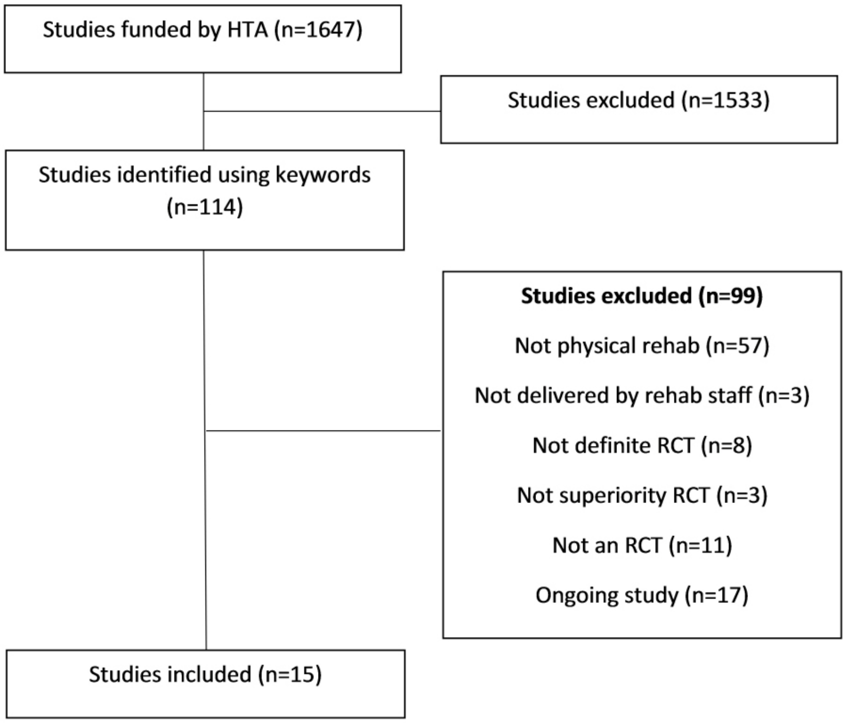

Figure 1 Study selection

Figure 1 Study selection

89x82mm (300 x 300 DPI)

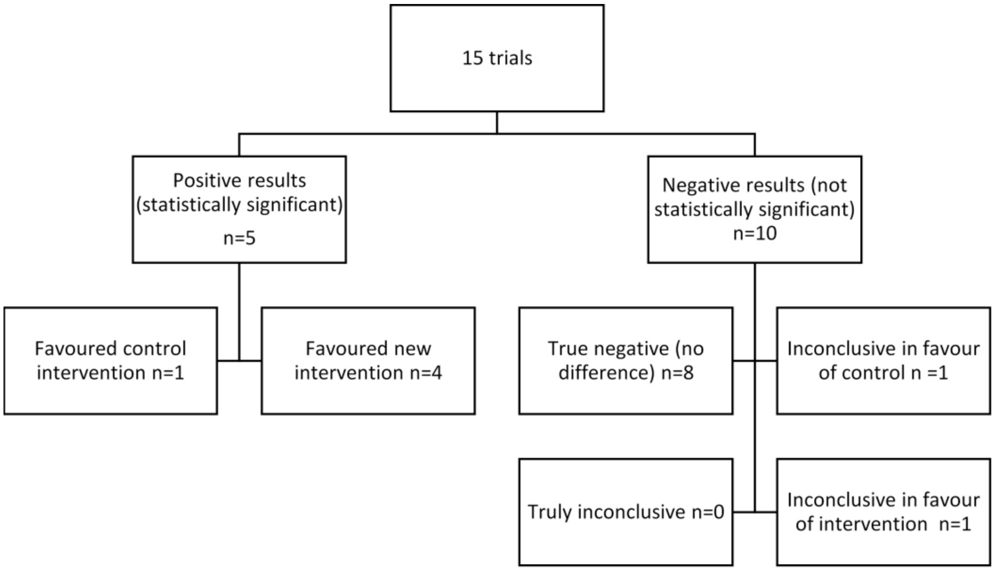

Figure 2 Classification of primary outcome

Figure 2 Classification of Primary Outcome

89x66mm (300 x 300 DPI)

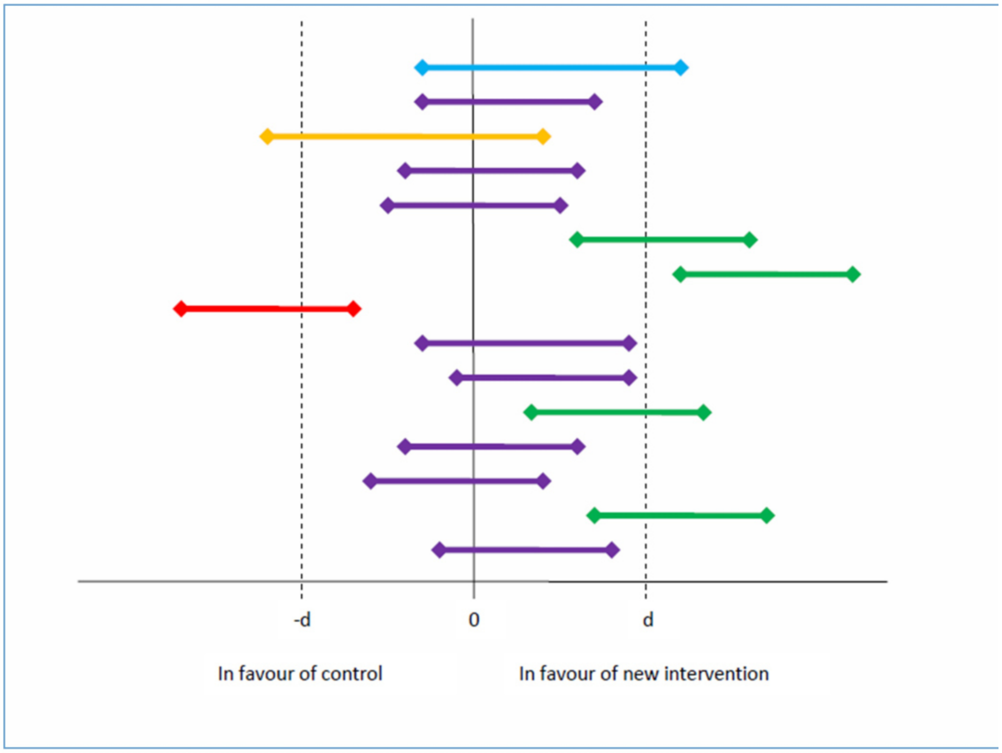

Figure 3 Treatment success of included trials based on 95% Confidence Interval and Minimal Clinically Important Difference from trial sample size calculation (d)

Key: *Green = statistically significantly in favour of intervention; Red = statistically significantly in favour of control; Blue = Inconclusive in favour of intervention; Yellow = Inconclusive in favour of control; Purple = True negative (no difference)*

Figure 3 Treatment success of included trials based on 95% Confidence Interval and Minimal Clinically Important Difference from trial sample size calculation (d)

87x89mm (300 x 300 DPI)
